# Supplementary material for: Index analysis: An approach to understand signal transduction with application to the EGFR signalling pathway
Source: PLoS Comput Biol. 2024 Feb 5;20(2):e1011777. doi: 10.1371/journal.pcbi.1011777 (PMC10868873; doi:10.1371/journal.pcbi.1011777)
Supplement: S1 Supplementary Material — (PDF) [file pcbi.1011777.s001.pdf]

S1 Supplementary Material

Index analysis: an approach to understand signal transduction with application to the EGFR signalling pathway

Jane Knöchel, Charlotte Kloft, Wilhelm Huisinga

Numerical computation of ir- and state classification indices

In the sequel, details are given for a single input state variable and a single output state variable—as a consequence, all indices are real-valued. The right hand side of the model system's ODEs, the function f, is defined in eq. (1) in the main text. Below, we specify the system of ODEs to be solved to determine each of the indices. For additional illustration, we use the simple reaction cycle model (see main text for details) with system of ODEs

$$\frac{\mathrm{d}}{\mathrm{d}t}A(t) = -k_a \cdot A(t); \qquad \frac{\mathrm{d}}{\mathrm{d}t}C(t) = k_{\mathrm{on}}A(t)B(t) - (k_{\mathrm{off}} + k_c)C(t)$$

$$\frac{\mathrm{d}}{\mathrm{d}t}B(t) = -k_{\mathrm{on}}A(t)B(t) + k_{\mathrm{off}}C(t); \qquad \frac{\mathrm{d}}{\mathrm{d}t}D(t) = k_{\mathrm{syn}}C(t) - k_{\mathrm{deg}}D(t),$$

initial conditions $(\mathrm{A},\mathrm{B},\mathrm{C},\mathrm{D})_0 = (0, 100, 5, 0)$ and input $u_0 = (2, 0, 0, 0)$. This results in

$$(\mathrm{A},\mathrm{B},\mathrm{C},\mathrm{D})(t_0) = (2, 100, 5, 0)$$

with $t_0 = 0$. The final time is either $T = 0.01$ or $T = 0.1$, depending on the scenario.

Input response indices The input-response index can be written in terms of two local sensitivity coefficients

$$\mathrm{ir}_k(t^*) = \left(\frac{1}{T}\int_{t^*}^{T} \mathcal{S}_{r,k}(t;t^*)^2 \mathrm{d}t\right)^{\frac{1}{2}} \cdot |\mathcal{S}_{k,i}(t^*;t_0)| \tag{S1}$$

with sensitivity coefficients

$$\mathcal{S}_{m,j}(t_2;t_1) = \big[\mathcal{S}(t_2;t_1)\big]_{m,j} = \left[\frac{\partial \Phi^{t_2,t_1}x}{\partial x}\bigg|_{x=x_{\mathrm{ref}}(t_1)}\right]_{m,j}. \tag{S2}$$

To compute the ir-indices in eq. (S1) numerically, we solved an extended system of ODEs to determine $\mathcal{S}(t_2;t_1)$ in eq. (S2):

$$\frac{\mathrm{d}}{\mathrm{d}t}x(t) = f\big(x(t);p\big) \tag{S3}$$

$$\frac{\mathrm{d}}{\mathrm{d}t}\mathcal{S}(t;t_1) = \frac{\partial f\big(x(t);p\big)}{\partial x} \cdot \mathcal{S}(t;t_1) \tag{S4}$$

on $t \in [t_1, t_2]$ with initial conditions $x(t_1) = x_{\mathrm{ref}}(t_1)$ and $\mathcal{S}(t_1;t_1) = \mathrm{Id}$. See, e.g., [1, 3.1] for details.

Environmental index To determine the environmental index of the kth state variable at time t^*, we solve the system of ODEs

$$\frac{\mathrm{d}x_{\mathrm{mod},j}}{\mathrm{d}t}(t) = f_j\big(x_{\mathrm{mod}}(t);p\big); \quad (j \neq k)$$

$$\frac{\mathrm{d}x_{\mathrm{mod},k}}{\mathrm{d}t}(t) = 0$$

for time $t \in [t^*, T]$ with initial conditions $x_{\text{mod}}(t^*) = x_{\text{ref}}(t^*)$. As a consequence, the kth state variables stays constant at the value $x_{\text{ref},k}(t^*)$ for $t \geq t^*$ in the modified system.
For the reaction cycle model and the kth state being C, the modified system of ODEs is

$$\frac{\mathrm{d}}{\mathrm{d}t}A(t) = -k_a \cdot A(t); \qquad \frac{\mathrm{d}}{\mathrm{d}t}C(t) = 0$$
$$\frac{\mathrm{d}}{\mathrm{d}t}B(t) = -k_{\text{on}}A(t)B(t) + k_{\text{off}}C(t); \qquad \frac{\mathrm{d}}{\mathrm{d}t}D(t) = k_{\text{syn}}C(t) - k_{\text{deg}}D(t)$$

Partial steady state index To determine the partial steady state index of the kth state variable at time t^*, we solve the system of ODEs

$$\frac{\mathrm{d}x_{\text{mod},j}}{\mathrm{d}t}(t) = f_j\big(x_{\text{mod}}(t); p\big); \quad (j \neq k)$$
$$0 = f_k\big(x_{\text{mod}}(t); p\big)$$

for time $t \in [t^*, T]$ with initial conditions $x_{\text{mod},j}(t^*) = x_{\text{ref},j}(t^*)$ for $j \neq k$. The initial condition of the kth state variable is implicitly defined by the algebraic condition $f_k(x_{\text{mod}}(t); p) = 0$. The above system is known as a differential-algebraic system of equations (DAE). It is of the general form

$$M\frac{\mathrm{d}}{\mathrm{d}t}x_{\text{mod}}(t) = f\big(x_{\text{mod}}(t); p\big) \tag{S5}$$

with so-called mass matrix M, defined as $M(j,j) = 1$ for $j \neq k$, $M(k,k) = 0$ and $M(j,l) = 0$ for $j \neq l$. There are many numerical integrators available that allow to integrate such DAEs.
For the reaction cycle model and the kth state being C, the modified system of ODEs is

$$\frac{\mathrm{d}}{\mathrm{d}t}A(t) = -k_a \cdot A(t); \qquad 0 = k_{\text{on}}A(t)B(t) - (k_{\text{off}} + k_c)C(t)$$
$$\frac{\mathrm{d}}{\mathrm{d}t}B(t) = -k_{\text{on}}A(t)B(t) + k_{\text{off}}C(t); \qquad \frac{\mathrm{d}}{\mathrm{d}t}D(t) = k_{\text{syn}}C(t) - k_{\text{deg}}D(t)$$

This can be rewritten as in eq. (S5):

$$\begin{pmatrix} 1 & & & \\ & 1 & & \\ & & 0 & \\ & & & 1 \end{pmatrix} \frac{\mathrm{d}}{\mathrm{d}t} \begin{pmatrix} A(t) \\ B(t) \\ C(t) \\ D(t) \end{pmatrix} = \begin{pmatrix} -k_a \cdot A(t) \\ -k_{\text{on}}A(t)B(t) + k_{\text{off}}C(t) \\ k_{\text{on}}A(t)B(t) - (k_{\text{off}} + k_c)C(t) \\ k_{\text{syn}}C(t) - k_{\text{deg}}D(t) \end{pmatrix}$$

Partially negligible index To determine the partially negligible index of the kth state variable at time t^*, we solve the system of ODEs

$$\frac{\mathrm{d}x_{\text{mod},j}}{\mathrm{d}t}(t) = f_j\big(\tilde{x}_{\text{mod}}(t); p\big); \quad (j \neq k)$$
$$\tilde{x}_{\text{mod},j}(t) = \begin{cases} x_{\text{mod},j}(t); & j \neq k \\ 0; & j = k \end{cases}$$

for time $t \in [t^*, T]$ with initial conditions $x_{\text{mod},j}(t^*) = x_{\text{ref},j}(t^*)$ for $j \neq k$.
For the reaction cycle model and the kth state being C, the modified system of ODEs is

$$\frac{\mathrm{d}}{\mathrm{d}t}A(t) = -k_a \cdot A(t);$$
$$\frac{\mathrm{d}}{\mathrm{d}t}B(t) = -k_{\text{on}}A(t)B(t); \qquad \frac{\mathrm{d}}{\mathrm{d}t}D(t) = -k_{\text{deg}}D(t)$$

As can be seen from the modified ODE, in this case the input would no longer be transmitted to the output. Thus, we would expect the pneg-index for C to be large. This can be seen from Figure 4B and 6B in the main text.

Completely negligible index To determine the completely negligible index of the kth state variable at time t^*, we solve the system of ODEs

$$\frac{\mathrm{d}x_{\mathrm{mod},j}}{\mathrm{d}t}(t) = \tilde{f}_j\big(\tilde{x}_{\mathrm{mod}}(t); p\big); \quad (j \neq k)$$
$$\tilde{x}_{\mathrm{mod},j}(t) = \begin{cases} x_{\mathrm{mod},j}(t); & j \neq k \\ 0; & j = k \end{cases}$$

for time $t \in [t^*, T]$ with initial conditions $x_{\mathrm{mod},j}(t^*) = x_{\mathrm{ref},j}(t^*)$ for $j \neq k$. Above, $\tilde{f}$ is determined from f by setting all reaction rate constants to zero that have the kth state variable as a product.
For the reaction cycle model and the kth state being C, the modified system of ODEs is

$$\frac{\mathrm{d}}{\mathrm{d}t}A(t) = -k_a \cdot A(t);$$
$$\frac{\mathrm{d}}{\mathrm{d}t}B(t) = 0; \qquad \frac{\mathrm{d}}{\mathrm{d}t}D(t) = -k_{\mathrm{deg}}D(t)$$

As for the partially negligible index, the input would no longer be transmitted to the output. Thus, we would expect the cneg-index for C to be large. This can be seen from Figure 4B and 6B in the main text.

References

1. Deuflhard P, Bornemann F. Scientific Computing with Ordinary Differential Equations. Springer, New York, 2002, 1st ed.
