# Supplementary material for: Index analysis: An approach to understand signal transduction with application to the EGFR signalling pathway
Source: PLoS Comput Biol. 2024 Feb 5;20(2):e1011777. doi: 10.1371/journal.pcbi.1011777 (PMC10868873; doi:10.1371/journal.pcbi.1011777)
Supplement: S2 Supplementary Material — (PDF) [file pcbi.1011777.s002.pdf]

S2 Supplementary Material

Index analysis: an approach to understand signal transduction with application to the EGFR signalling pathway

Jane Knöchel, Charlotte Kloft, Wilhelm Huisinga

Pseudocode of numerical computation of ir- and state classification indices

1: **Setting:** Input = ith state., output = oth state; indices to be determined for kth state; the numerical integrator should allow for the solution of DAEs and preferably also allows for a non-negativity constraint

2: **Reference solution & controllability coefficient:** Solve the extended model

$$\frac{\mathrm{d}}{\mathrm{d}t}x_{\mathrm{ref}}(t) = f\big(x_{\mathrm{ref}}(t);p\big) \qquad \frac{\mathrm{d}}{\mathrm{d}t}\mathcal{S}(t;t_0) = \frac{\partial f\big(x_{\mathrm{ref}}(t);p\big)}{\partial x}\cdot\mathcal{S}(t;t_0)$$

on $t\in[t_0,T]$ with initial conditions $x_{\mathrm{ref}}(t_0)=x_0+u_0$ and $S(t_0;t_0)=\mathrm{Id}$. Define

$$\text{tspan} \leftarrow \text{set of integration time points} \qquad\qquad (\text{set of } t^* \text{ values})$$

and set for each $t^*\in$ tspan

$$c_k(t^*) \leftarrow \big|S_{k,i}(t^*;t_0)\big| \qquad\qquad (\text{controllability coefficient})$$

3: **Observability coefficient:** For each $t^*\in$ tspan, solve the extended model

$$\frac{\mathrm{d}}{\mathrm{d}t}x(t) = f\big(x(t);p\big) \qquad \frac{\mathrm{d}}{\mathrm{d}t}\mathcal{S}(t;t^*) = \frac{\partial f\big(x(t);p\big)}{\partial x}\cdot\mathcal{S}(t;t^*)$$

on $t\in[t_*,T]$ with initial conditions $x(t^*)=x_{\mathrm{ref}}(t^*)$ and $S(t^*,t^*)=\mathrm{Id}$. Use the trapezoidal rule to determine

$$o_k(t^*) \leftarrow \left(\frac{1}{T}\int_{t^*}^{T} S_{o,k}(T;t^*)^2\mathrm{d}t\right)^{1/2} \qquad\qquad (\text{observability coefficient})$$

4: **Input-response index:** For each $t^*\in$ tspan, define

$$\mathrm{ir}_k(t^*) \leftarrow o(t^*)c(t^*) \qquad\qquad (\text{ir-index})$$

$$\mathrm{nir}_k(t^*) \leftarrow \frac{\mathrm{ir}_k(t^*)}{\sum_{j=1}^{n}\mathrm{ir}_j(t^*)} \qquad\qquad (\text{normalised ir-index})$$

5: **State classification indices (env, pss, pneg, cneg):** Solve the modified ODE system on $t\in[t_*,T]$ with modified initial conditions $x_{\mathrm{mod}}(t^*)$. The details depend on the specific state classification index; see previous section for details. Use the trapezoidal rule to determine

$$Z(t^*) \leftarrow \int_{t^*}^{T}|x_o(t)|^2\mathrm{d}t \qquad\qquad (\text{normalisation constant})$$

$$\mathrm{mod}_k(t^*) \leftarrow \left(\frac{1}{Z(t^*)}\int_{t^*}^{T}\big([x_{\mathrm{ref}}(s)-x_{\mathrm{mod}}(s)]_o\big)^2\mathrm{d}s\right)^{1/2} \qquad\qquad (\text{state classification index})$$

For some combinations of modifications & states, the numerical integrator might not be able to solve the modified system (blow up, unable to find consistent initial conditions for pss case, etc). In this case, assign 'NaN' to the corresponding index and proceed.
