# Supplementary material for: Index analysis: An approach to understand signal transduction with application to the EGFR signalling pathway
Source: PLoS Comput Biol. 2024 Feb 5;20(2):e1011777. doi: 10.1371/journal.pcbi.1011777 (PMC10868873; doi:10.1371/journal.pcbi.1011777)
Supplement: S3 Supplementary Material — (PDF) [file pcbi.1011777.s003.pdf]

S3 Supplementary Material

Index analysis: an approach to understand signal transduction with application to the EGFR signalling pathway

Jane Knöchel, Charlotte Kloft, Wilhelm Huisinga

How to set up a new model for Index Analysis

Setting up a new model is easy and proceeds along the steps below. The `SimpleReactionCycle` folder and files serves as a starting point. The model is described in the first part of the Results in the main manuscript.

Copy the folder `SimpleReactionCycle` and rename it with a meaningful `<ProjectName>`; change into the renamed directory and then into the subfolder `modelspecification`; adapt the following files as outlined below (and rename appropriately by replacing `SimpleReactionCycle_<Filename>.m` with `<YourModelName>_<Filename>.m`)

%%% -
For all template files provided, you only need to adapt the code between the two dashed lines; do not modify the code outside those line (except for the name of the file in the line starting with `function`).
%%% -

1. `SimpleReactionCycle_indexing.m`: provide the names of all state variables in the structure `I.nmstate` and the names of all parameters in `I.nmpar`.

2. `SimpleReactionCycle_initialvalues.m`: provide the initial values of the pre-stimulus state of the system; the pre-stimulus state does not include the stimulus (input), which is zero and specified in the `SimpleReactionCycle_model_set_up_details.m`. In the template code, A is the stimulus/input and C is the output

3. `SimpleReactionCycle_parameters.m`: provide the values for all parameters

4. `SimpleReactionCycle_ode.m`: provide the ordinary differential equations (ODEs);

5. `SimpleReactionCycle_species2params.m`: provide for each state the parameter indices of all reactions that involve that specific state; the information is used for the cneg index.

6. `SimpleReactionCycle_model_set_up_details.m`: provides all the final details to simulate the model and compute the indices; provide the model name (i.e. `YourModelName`, see above), specify the input state, the output state and the simulation time span; in addition, you can specify different scenarios (in the template code, different values for a parameter); if a jacobian or legend labels are provided (see below), set corresponding binary variables to `true`.

7. `SimpleReactionCycle_odejac.m` (optional): provide the jacobian of the system of ODEs; if no jacobian is provided then the ODE solver uses a standard numerical approximation. Note: errors in jacobians for larger systems are easily made, in particular, if it is a larger system and done manually; do compare your results with the case, where the jacobian is numerically determined by MATLAB.

8. `SimpleReactionCycle_legendlabels.m` (optional): sometimes the names in the code are not easily readable when used in a figure legend; here one can provide for each state a name used in figure legend.

Finally, exit the directory `modelspecification` to the parent `<ProjectName>` directory. The final step is to adapt the main script `SimpleReactionCycle_MAIN.m` (rename meaningfully to `<YourModelName>_MAIN.m`). Specify the model name and the scenario (see `SimpleReactionCycle_model_set_up_details.m` above) and the desired analysis plots.

You are now ready to run the main script `<YourModelName>_MAIN.m` !
