# Supplementary material for: Index analysis: An approach to understand signal transduction with application to the EGFR signalling pathway
Source: PLoS Comput Biol. 2024 Feb 5;20(2):e1011777. doi: 10.1371/journal.pcbi.1011777 (PMC10868873; doi:10.1371/journal.pcbi.1011777)
Supplement: S4 Supplementary Material — Section S4.1. Index analysis for parallel pathway model. Section S4.2. Index analysis for enzyme substrate model. (PDF) [file pcbi.1011777.s004.pdf]

S4 Supplementary Material

Index analysis: an approach to understand signal transduction with application to the EGFR signalling pathway

Jane Knöchel, Charlotte Kloft, Wilhelm Huisinga

Index analysis for additional small-scale illustrative model systems

S4.1 Index analysis for parallel pathway model

Considered the following simple parallel pathway reaction model sketched in Figure I. In the model, the signal A activates S into B and C, which in turn activate D. Furthermore, B and C may be deactivated to S again. In this example, A is considered the input and D the response variable. We study two different scenarios given by the parameters in Table I.

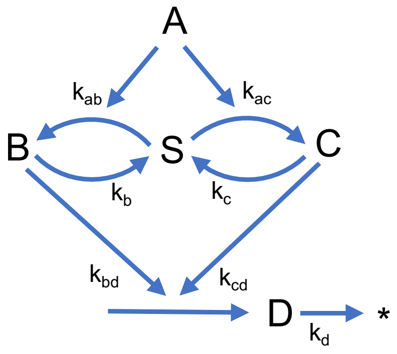

Figure I. Reaction network of the parallel pathways model.

Table I. Model specification for the parallel pathway model. For the two scenarios of the model in Figure I, the parameter values are stated below. The time span was $t \in [0, 0.05]$ min, the input A, and the response D. The initial conditions were $(A_0, B_0, C_0, D_0) = (20, 0, 0, 0)$ nM, in addition to S_0 (in nM) specified below. Units: $k_{ab}, k_{ac}, k_{bd}, k_{cd}$ in 1/nM/min; all other reaction rate constants in 1/min.

Scenario	S_0	k_{ab}	k_{ac}	k_b	k_c	k_{bd}	k_{cd}	k_d
no crosstalk	3000	50/300	1/300	5	500	0.4	11	25
with crosstalk	10	50	1	5	500	0.4	11	25

Figure IIA shows the time course of the state variables, while Figure IIB shows the normalised ir-indices. The dynamic importance of state variables changes substantially during the first 0.01 min: While the input A quickly falls below the threshold, S and B substantially increase and then decrease still within the first 0.01 min. During the same time, the response D steady increasing. Only the state variable C stays far below the threshold of 10 %. Consequently, we would consider all state variables except C as dynamically important.

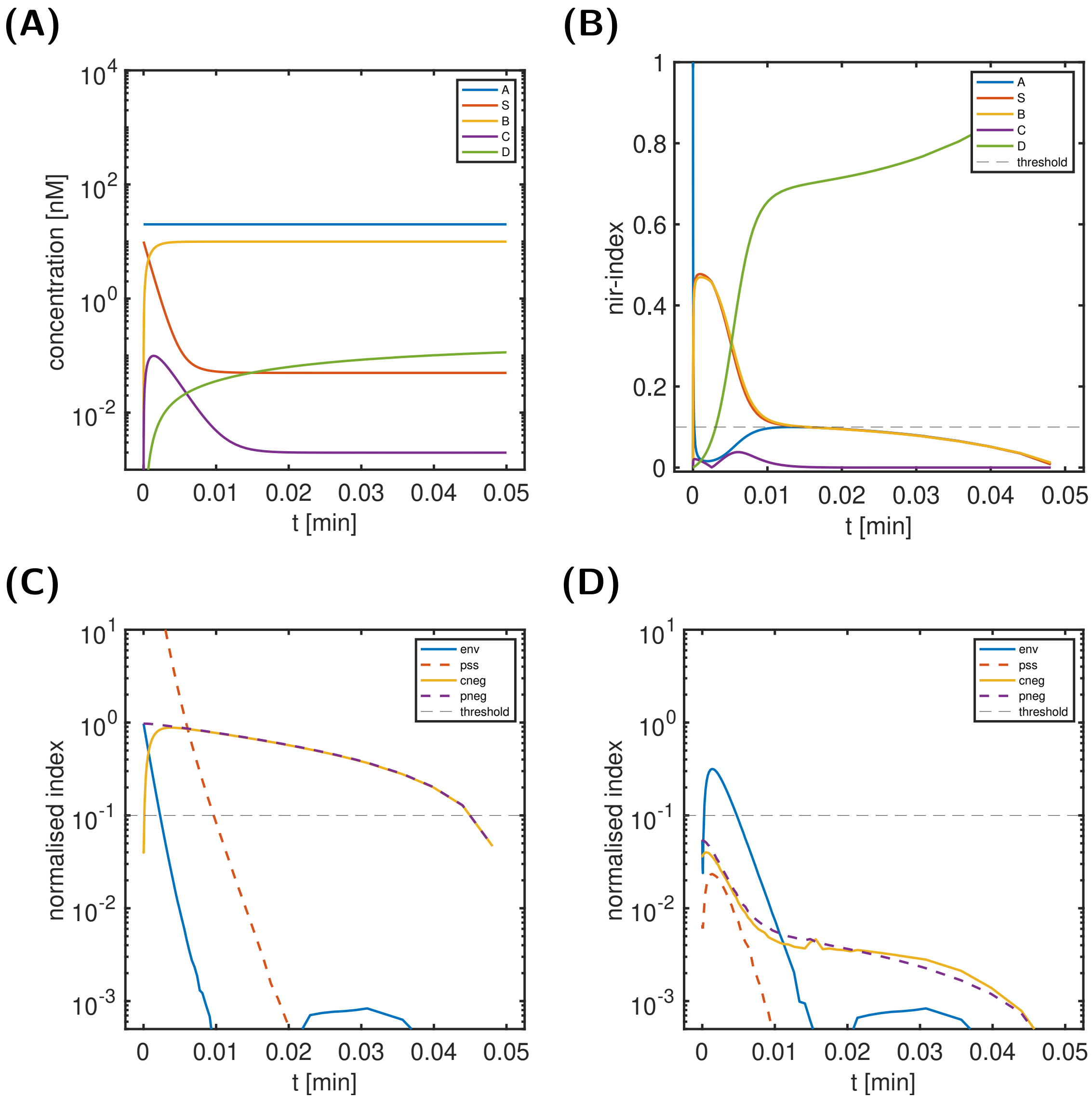

Figure II. Scenario 'with crosstalk' of the parallel pathways model: time course of state variables and normalised indices. A: Time course of state variables and B: normalized ir-indices. C: State classification indices for states B and D: for C.

The bottom graphics of Figure II show the state classification indices for two states, B (C) and C (D). Again, a state classification index below the threshold is considered indicative of the corresponding index classification type (environmental, in partial steady state, negligible). From the normalised ir-index we would expect for B none of the state-classification indices below the threshold. This is indeed the case, as shown in the Figure IIC. Figure IID gives further insight on the potential nature of the state C. All but the env-index are below the threshold.

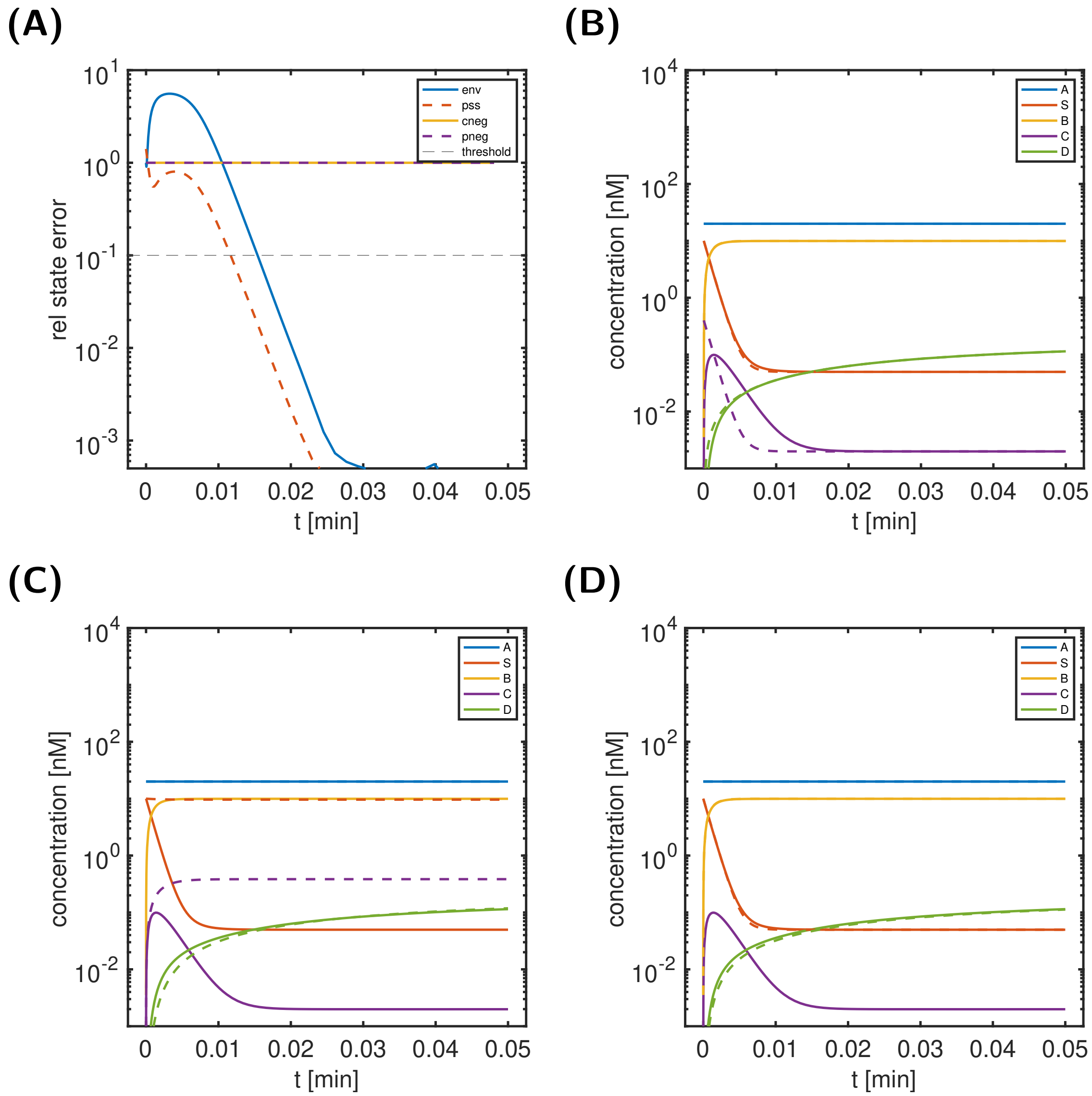

Figure III. Scenario 'with crosstalk' of the parallel pathways model: Relative state approximation error and modified dynamics. A: Relative state approximation errors for C. B: Comparison of reference dynamics (solid lines) and modified dynamics from $t^* = 0$ (dashed lines) for C in partial steady state or D: completely neglected. C: Comparison of reference dynamics (solid lines) and modified dynamics from $t^* = 0$ (dashed lines) for B completely neglected.

For a final classification of C, also the relative state approximation error (shown in Figure III) is taken into account. However, none of the relative state approximation errors is constantly below the threshold. Hence, C is neither classified as environmental nor in partial steady state. To illustrate the latter, Figure IIIB shows the reference solution and the solution of the modified system (dashed lines) with C in partial steady state. While the approximation error for the output looks reasonable (as we would expect from the state classification index), the approximation error for the state itself is substantial (as we would expect from the relative state approximation error, Figure IIIA: the red dashed line at $t = 0$ is even above 10^0).

Finally, we analysed the impact of completely neglecting one of the two pathways, i.e., B or C. From the

normalised ir-indices (Fig. IIC&D), we infer that for both, B and C, the cneg-index at $t = 0$ is below the threshold. Therefore, we would expect an acceptable approximation of the reduced model with B or C completely neglected (from time $t = 0$ on). Figure IIIC&D shows the reference solution and the solution of the modified system (dashed lines) with B (left) or C (right) completely neglected. The impact on the response D is in both cases small—in line with the normalised ir-index at $t = 0$ (Fig. IIC&D). Looking at the normalised cneg-index (solid yellow line) of the entire time span, however, we see a marked difference between the two state variables: While for C, the cneg-index is below the threshold for the entire time span, for B it is only below the threshold at the very beginning and at the very end. If we would evolve the reference system up to, e.g., time $t = 0.01$ and only then completely neglect B, we would see a relatively large error in the response variable (yellow line almost 10^0), while doing the same for C, we would see almost no error in the response variable (yellow line even below 10^{-2}). This is an important difference! The fact that the cneg-index for B is only low initially indicates that the system is capable of compensating for a complete loss of B, if it happens from the onset of the signal, while at later times, it is not able to compensate without a marked impact on the response D. In contrast, the system is able to compensate for a loss of C at any time, from signal onset at $t = 0$ to the very end.

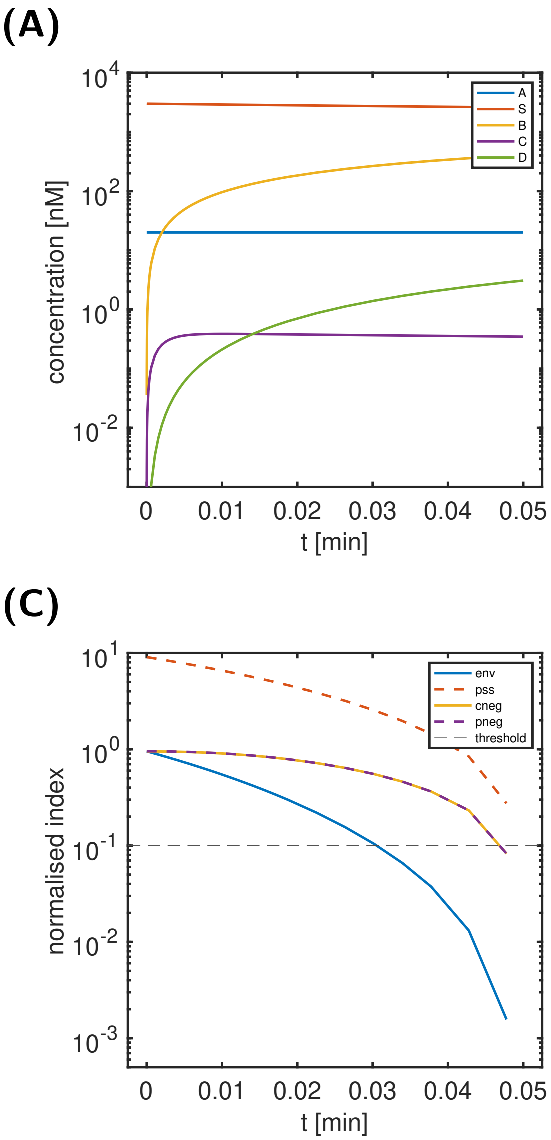

Figure IV. Scenario no crosstalk of the parallel-pathways model: A: Time course of state variables and B: normalised ir-indices. C: state classification indices for B and D: for C.

In the above scenario, the crosstalk is caused by the competition for the limited resource of S. Due to the specific parameter values, S is much more strongly activated to B than to C. As a consequence, C can be completely neglected with only minor impact on the response. If, however, B is missing, then C is able to activate D with a very similar profile. This feature disappears, if there is no resource limitation of S, i.e., no crosstalk: Figures IVA&B shows the concentration-time profiles and the normalised ir-indices.
Figures IVC&D shows the state classification indices for B (left) and C (right). In contrast to the scenario with crosstalk, for B also the cneg index is close to 1 initially. Hence, completely neglecting B from $t = 0$ should have a large impact on the response. This can be see from Figure VA, which shows the reference solution and the solution of the modified system (dashed lines) with B completely neglected. Hence, C is not able to activate D in a similar matter as B does, if the latter is missing. The impact of lacking C, however, does not change quantitatively between the scenarios with/without crosstalk, as can be inferred from Figure IVD, and Figure VB.

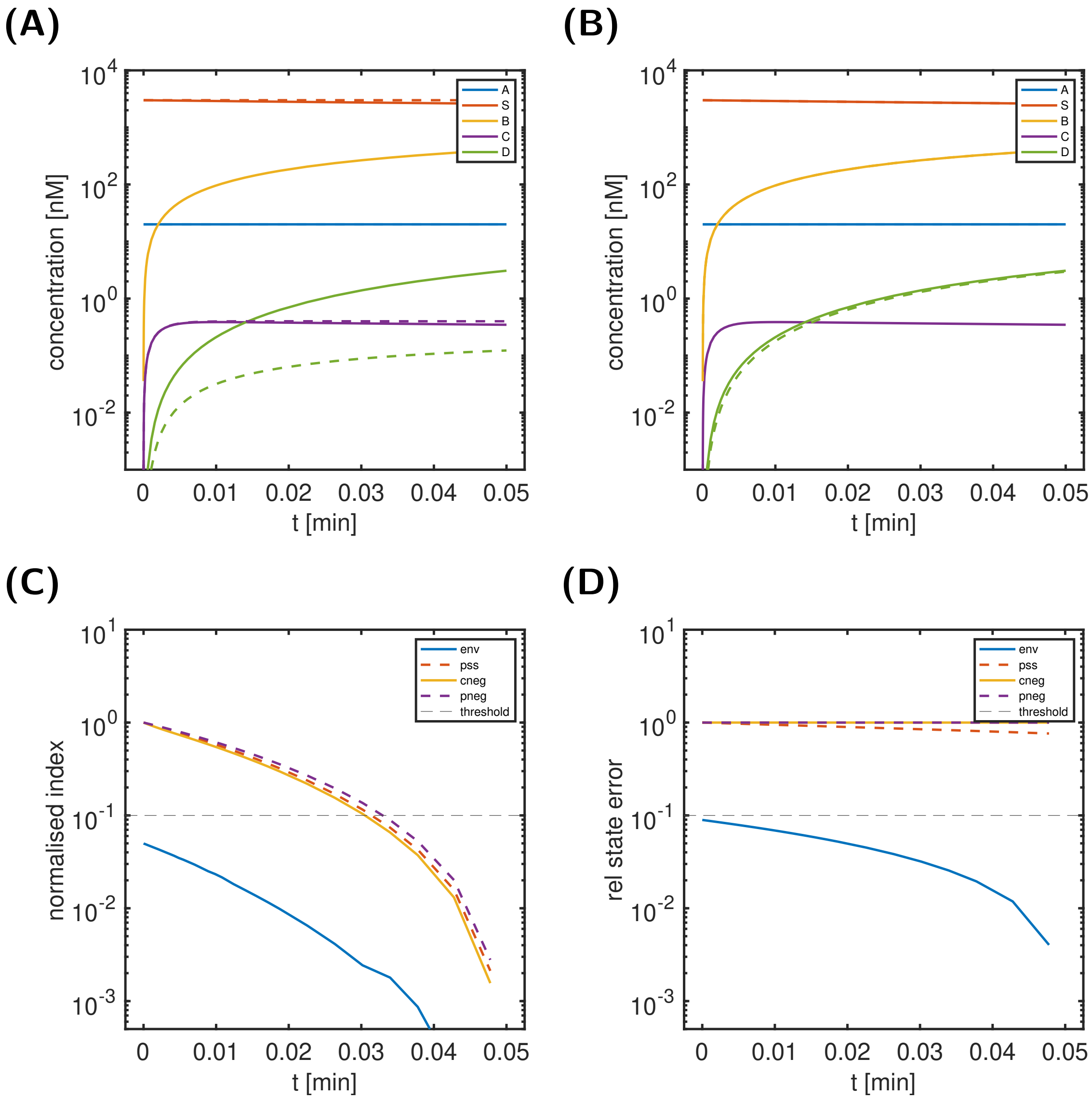

Figure V. Scenario no crosstalk of the parallel-pathways model: A: Comparison of reference dynamics (solid lines) and modified dynamics from $t^* = 0$ (dashed lines) for B and B: for C being completely neglected. C: state classification indices and D: relative state approximation errors for S.

Finally, from bottom panel of Figure V, we conclude that S can be considered as environmental, since considering S being constant does have only negligible impact on the response (bottom left, blue line) as well as on the state itself (bottom right, blue line). This is in line with our expectation, since the presence or absence of crosstalk is linked to S being a limited or abundant resource, respectively (see also S_0 in Table I).

S4.2 Index analysis for enzyme substrate model

Considered the simple enzyme kinetics model sketched in Figure VI. In the model, the protein A is transformed into substrate S, which subsequently forms a complex C with an enzyme E. The complex either dissociates or is catabolised to a product P. In this example, A is considered the input and P the response variable. We study two different scenarios given by the parameters in Table II. The parameters sets were chosen to illustrate the application to this well-known example and indicate extensions to account for conservation laws.

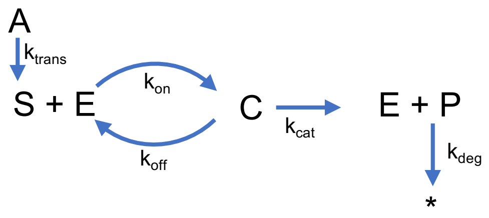

Figure VI. Reaction network of the enzyme kinetics model.

Table II. Model specification for the enzyme kinetics model. For the two scenarios of the model in Figure VI, the parameter values are stated below. The time span was $t \in [0, 30]$ min, the input A, and the response P. The initial conditions were $(\mathrm{A}_0, \mathrm{S}_0, \mathrm{C}_0, \mathrm{P}_0) = (50, 0, 0, 0)$ nM, in addition to E_0 (in nM) specified below. Units: k_{on} in 1/nM/min; all other reaction rate constants in 1/min.

Scenario	E_0	k_{on}	k_{off}	k_{cat}	k_{trans}
1	25	1	900	5	0.5
2	2	0.2	10	1	0.1

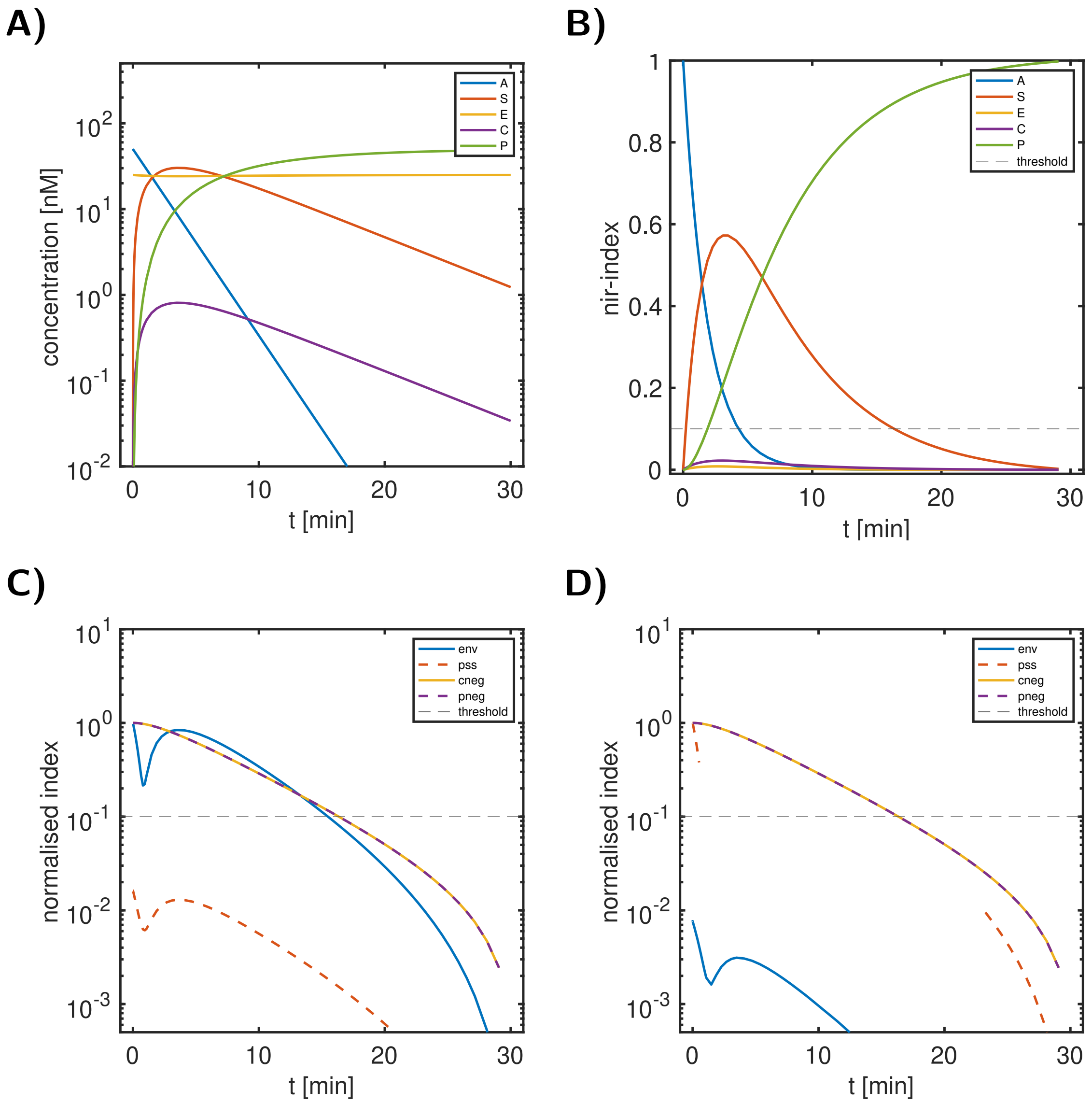

Figure VII. Scenario 1 of the enzyme kinetics model: time course of state variables and normalised indices . A: Time course of state variables and B: normalized ir-indices. C: State classification indices for state C and D: for state E.

Figure VIIA&B shows the time course of the state variables (left) and the normalised ir-indices (right). Except for E and C, all state variables are at some point in time above the threshold and are thus classified as dynamically importance. Figure VIIC&D depicts the state classification indices for C and E. We hypothesise that C is in partial steady state and E is environmental.

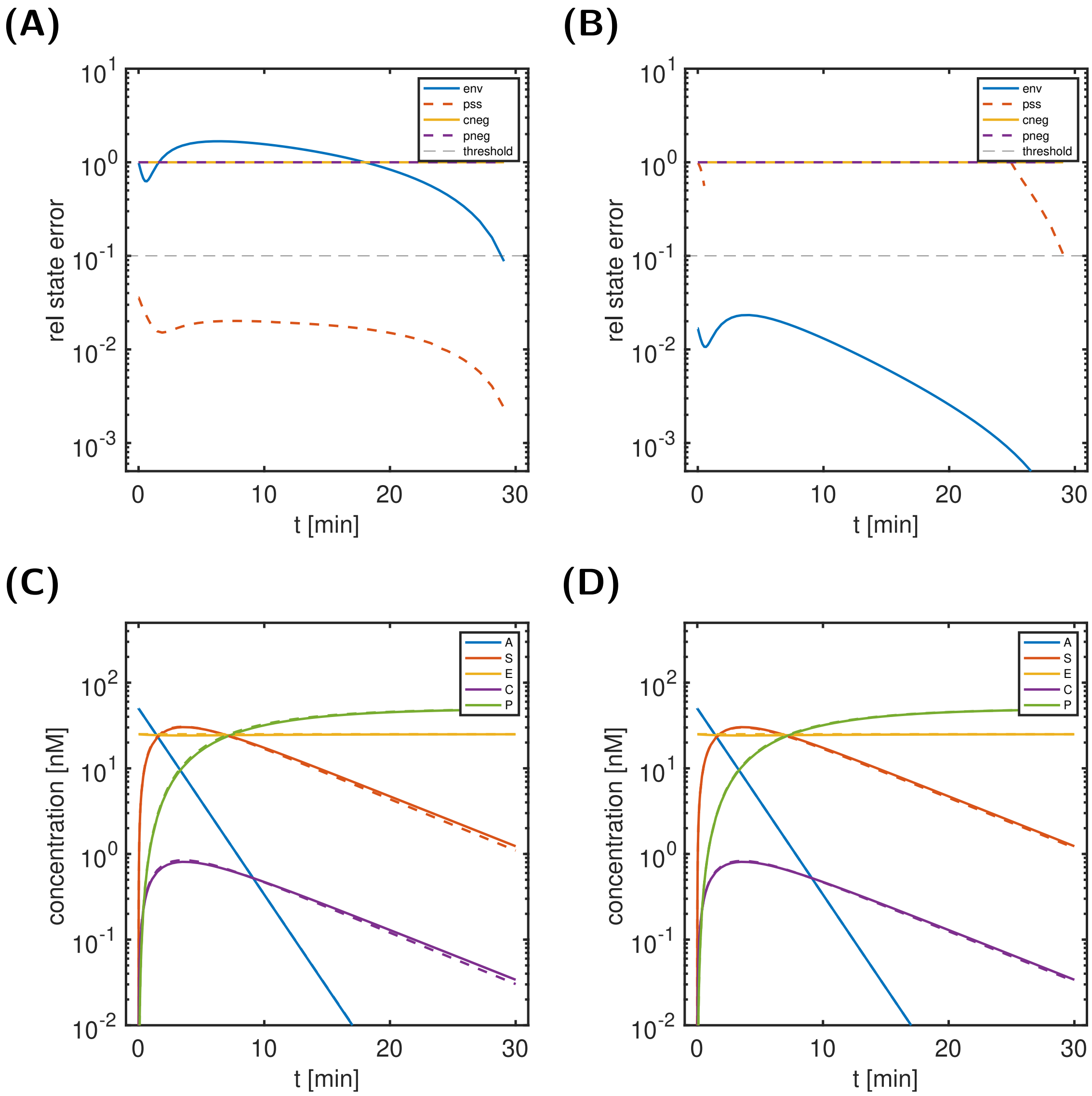

Figure VIII. Scenario 1 of the enzyme kinetics model: Relative state approximation error and modified dynamics . A: Relative state approximation errors for C and B: for E. C: Comparison of reference dynamics (solid lines) and modified dynamics from $t^* = 0$ (dashed lines) for C in partial steady state and D: for E as environmental state.

Figure VIII confirms our hypothesized classification. For C (left column) and E (right column), the corresponding relative state approximation errors (top) are below the threshold. A comparison of the reference solution and the solution of the modified system (dashed lines) with C in partial steady state or E environmental, respectively show the very good approximation quality.

The enzyme kinetic example is THE example to illustrate the quasi-steady state or Michaelis-Menten approximation. In its derivation, the conservation of enzyme is typically exploited. In scenario 1, this conservation is not needed to yield good approximation results for the partial steady state approximation of C. In scenario 2, however, the conservation law has to be exploited to obtain high quality approximations. Exploiting a conservation law for E in combination with the partial steady state approximation for C is identical to first transforming the system of ODEs into an equivalent system of ODEs with the equation for E replaced by Etot, i.e., the sum of the equations for E and C (i.e., the conserved species). Note that the conservation law still allows to recompute the free enzyme concentration as E=Etot-C. Then, the partial steady state approximation for C can be applied as usual. Transforming a system of ODEs in the described way is nothing else then lumping the states E and C into a new state representing Etot. In the following, we briefly demonstrate that the indices can be extended to scenarios corresponding to the Michaelis-Menten approximation (scenario 2). An extension of the index analysis approach to conservation laws is, however, beyond the scope of the present article.

Figure IXA&B shows in the top panel the reference solution (left) and the normalised ir-indices (right) for scenario 2. We infer that all states have to be considered dynamically important. Consequently, assuming C in partial steady state (Figure IXC) results in large approximation errors. In contrast, transforming the system of ODEs as described above allows us to account for the conservation law E+C = const. Assuming now C to be in partial steady state yields a very good approximation, as the comparison of reference dynamics (solid lines) and modified dynamics from $t^* = 0$ (dashed lines) shows (Figure IXD). The resulting approximation is identical to the common Michaelis-Menten approximation for S with $V_{\max} = 2\,\mathrm{nM/min}$ and $K_M = 55\,\mathrm{nM}$.

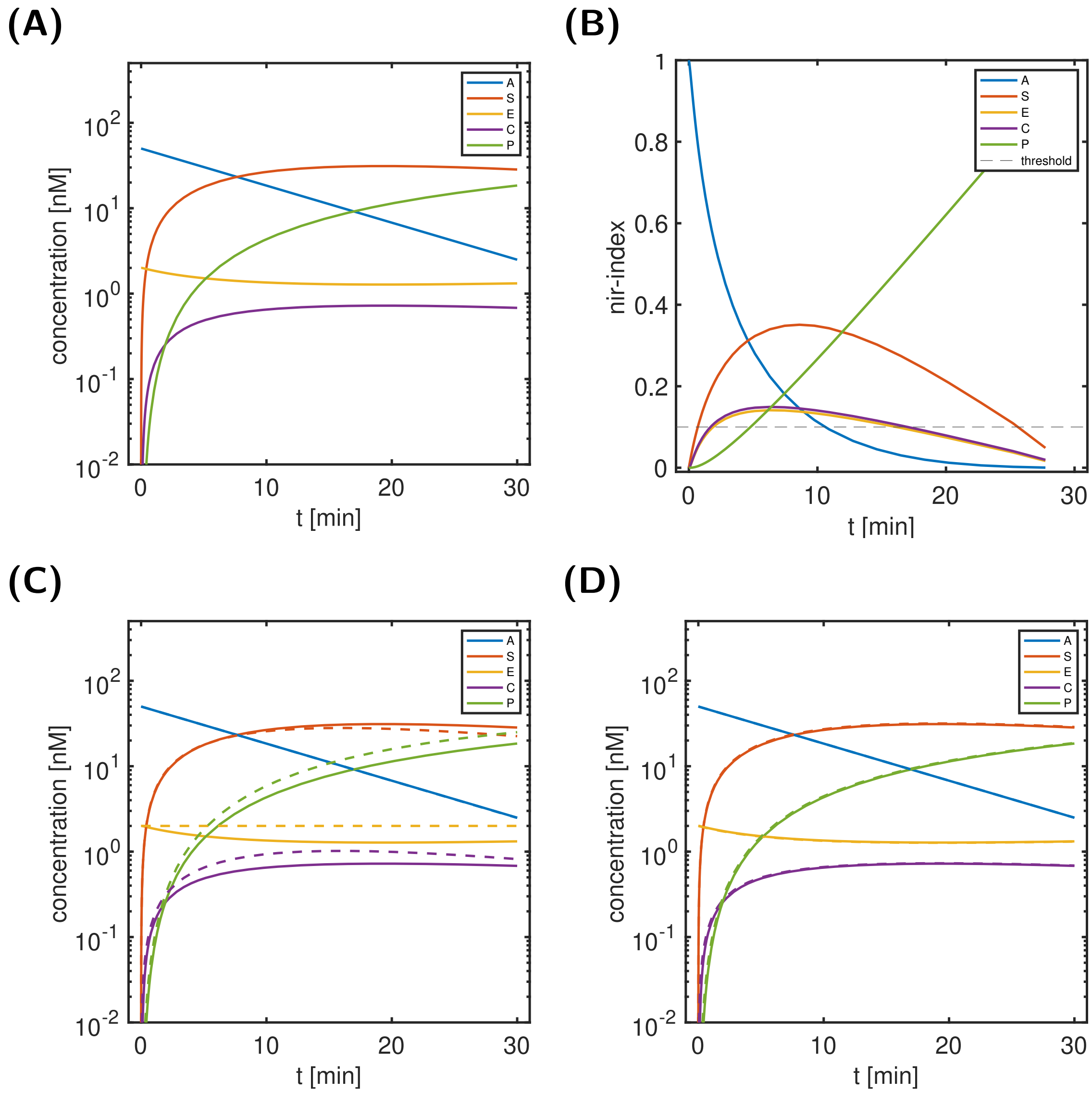

Figure IX. Scenario 2 of the enzyme kinetic model: A: Time course of state variables and B: normalised ir-indices. C: Comparison of reference dynamics (solid lines) and modified dynamics from $t^* = 0$ (dashed lines) for C in partial steady state. D: Comparison of reference dynamics (solid lines) and modified dynamics from $t^* = 0$ (dashed lines) with conservation law E+C in addition to C in partial steady state.

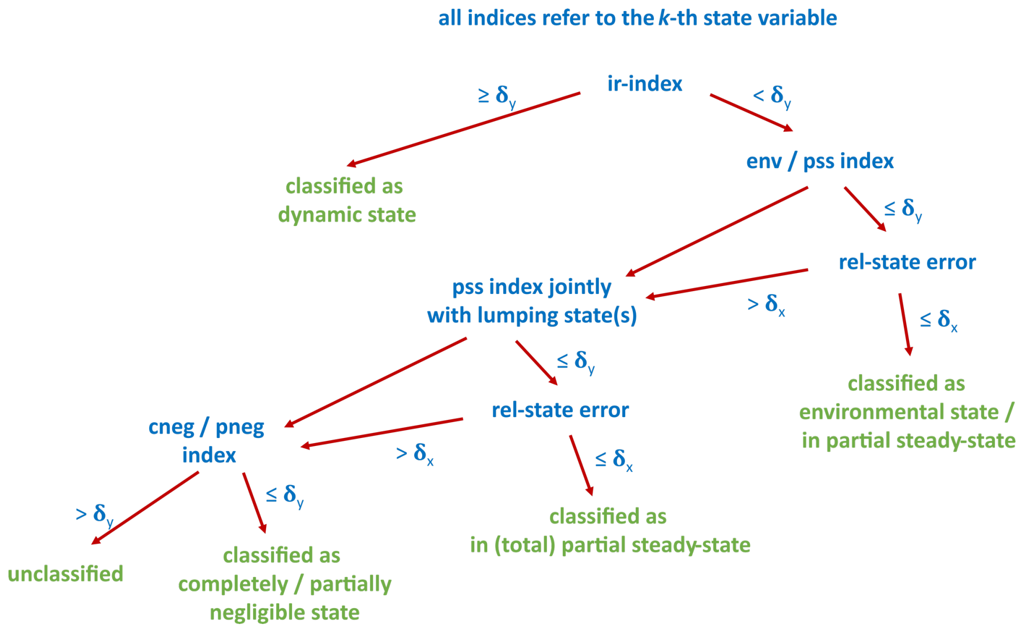

Figure X. Decision tree for state classification based on indices including lumping
