# Supplementary material for: Index analysis: An approach to understand signal transduction with application to the EGFR signalling pathway
Source: PLoS Comput Biol. 2024 Feb 5;20(2):e1011777. doi: 10.1371/journal.pcbi.1011777 (PMC10868873; doi:10.1371/journal.pcbi.1011777)
Supplement: S5 Supplementary Material — Fig I. Simplified illustration of EGFR signalling cascade. Fig II. EGFR signalling network prior to a stimulus. An illustration of state variables that are not in steady state prior to any EGF stimulus. For details on the model, see Material Section in the main article. (PDF) [file pcbi.1011777.s005.pdf]

S5 Supplementary Material

Index analysis: an approach to understand signal transduction with application to the EGFR signalling pathway

Jane Knöchel, Charlotte Kloft, Wilhelm Huisinga

EGFR system

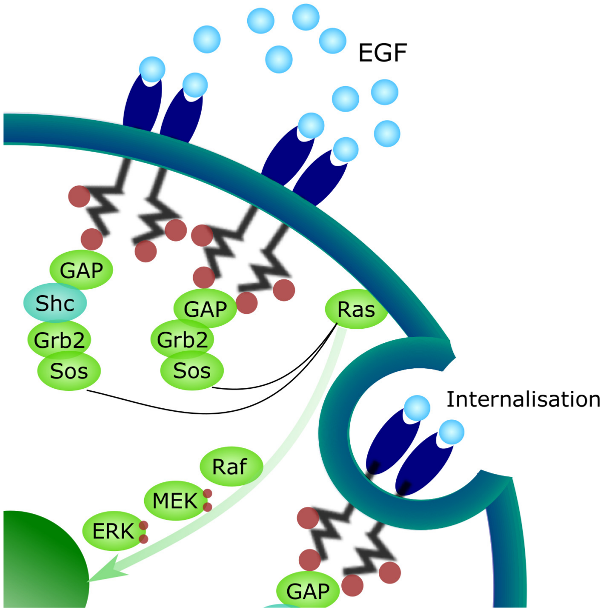

Figure I. Simplified illustration of EGFR signalling cascade.

The EGFR signalling network is activated by the binding of the ligand EGF to its receptor (EGFR) and results in a transient double-phosphorylation of the extracellular signal-regulated kinase (ERK-PP); see Figure I.

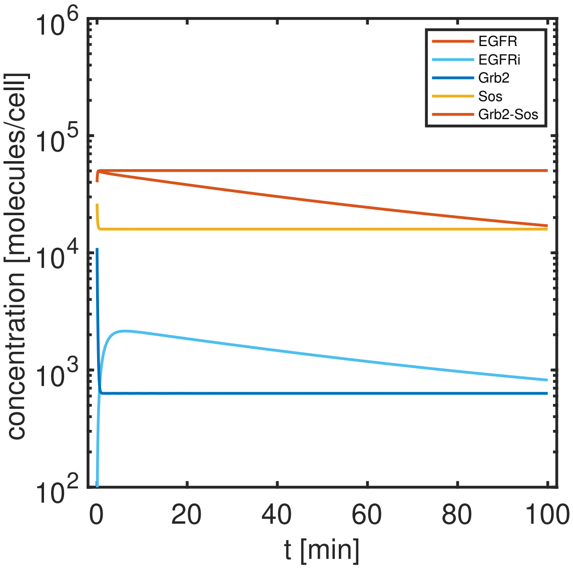

Figure II. EGFR signalling network prior to a stimulus. An illustration of state variables that are not in steady state prior to any EGF stimulus. For details on the model, see Material Section in the main article.
