# Supplementary material for: Index analysis: An approach to understand signal transduction with application to the EGFR signalling pathway
Source: PLoS Comput Biol. 2024 Feb 5;20(2):e1011777. doi: 10.1371/journal.pcbi.1011777 (PMC10868873; doi:10.1371/journal.pcbi.1011777)
Supplement: S6 Supplementary Material — Fig I. Schematic with state classification of the signal transduction network focussing on the Shc-independent pathway, including the state variables with large maximum input-response index (light blue, see Fig 4D), environmental state variables (purple), state variables in partial-steady state (green) and further state variables (dark blue). States being part of the membrane-bound and internalised pathway are coloured orange in panel (B). The red boxes mark the input and output state variables. Note that the difference to the Shc-dependent pathway is the absence of Shc (adaptor protein between GAP and Grb2). Fig II. Number of normalised ir-indices above the threshold of 10% as a function of time. Fig III. Sum of contr- and obs-indices evolving over time. Left: Sum of contr-indices over time, showing three phases: initial sharp peak (0–0.3 min), second prolonged peak (0.3–3 min), and a slow, still incomplete recovery period (3–100 min). Right: Sum of obs-indices over time, showing three phases: initial sharp decline (0–1.5 min), marginal increase (1.5–20 min), and a strong decline (20–100 min). Note the very different scales on the y-axis. Fig IV. Relative state approximation errors for Phosphatase1. Fig V. Time course of important species related to Phosphatase2. Table I. Normalised input-response indices for the EGFR system; sorted according to their maximum. (PDF) [file pcbi.1011777.s006.pdf]

Supplementary Material

Index analysis: an approach to understand signal transduction with application to the EGFR signalling pathway

Jane Knöchel, Charlotte Kloft, Wilhelm Huisinga

Additional figures and tables for the index analysis of the EGFR system

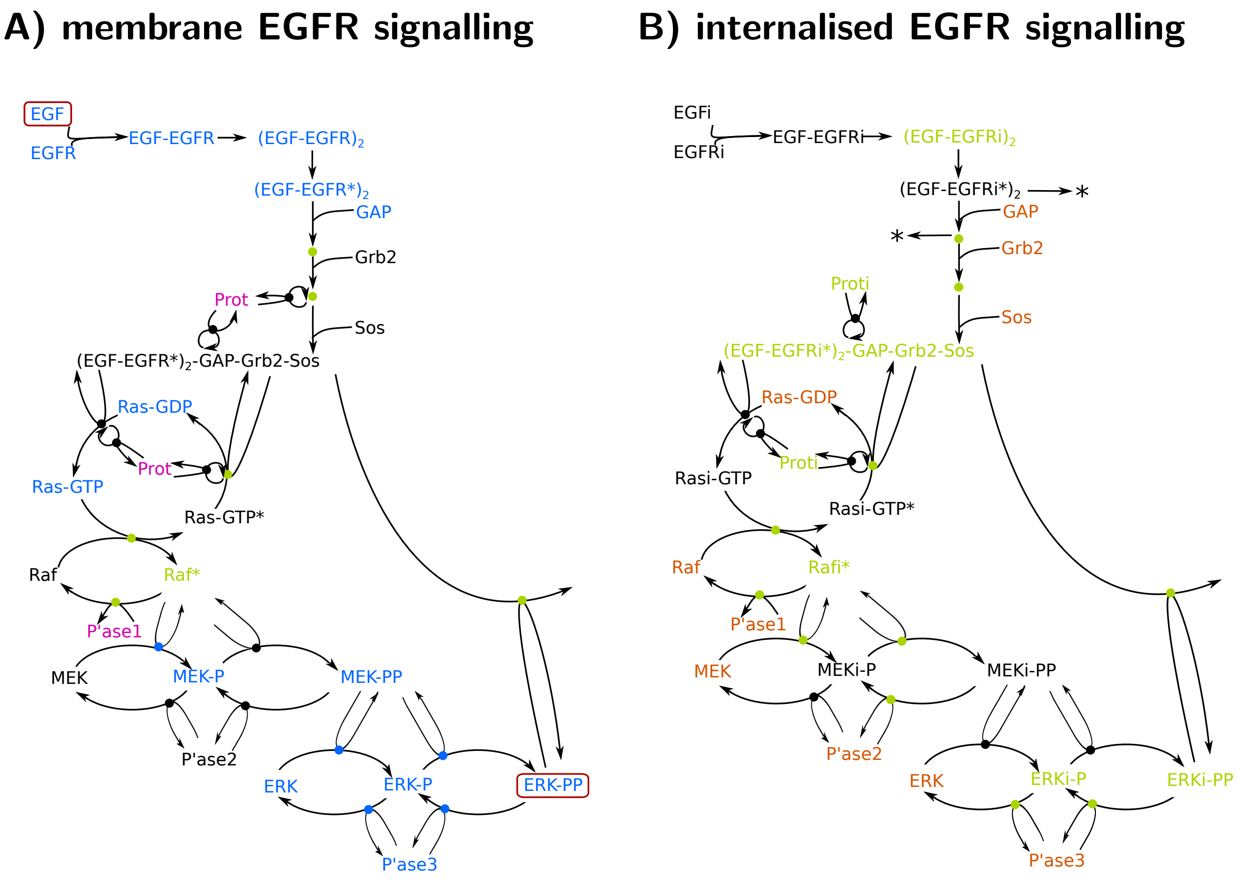

Figure I. Schematic with state classification of the signal transduction network focussing on the Shc-independent pathway, including the state variables with large maximum input-response index (light blue, see Figure 4D), environmental state variables (purple), state variables in partial-steady state (green) and further state variables (dark blue). States being part of the membrane-bound and internalised pathway are coloured orange in panel (B). The red boxes mark the input and output state variables. Note that the difference to the Shc-dependent pathway is the absence of Shc (adaptor protein between GAP and Grb2).

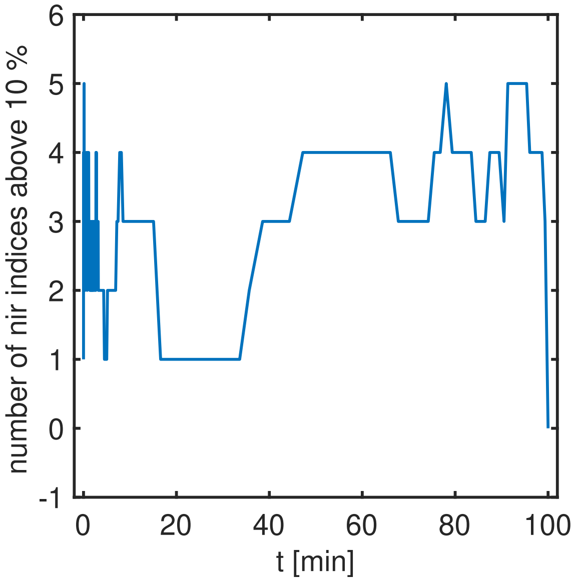

Figure II. Number of normalised ir-indices above the threshold of 10% as a function of time.

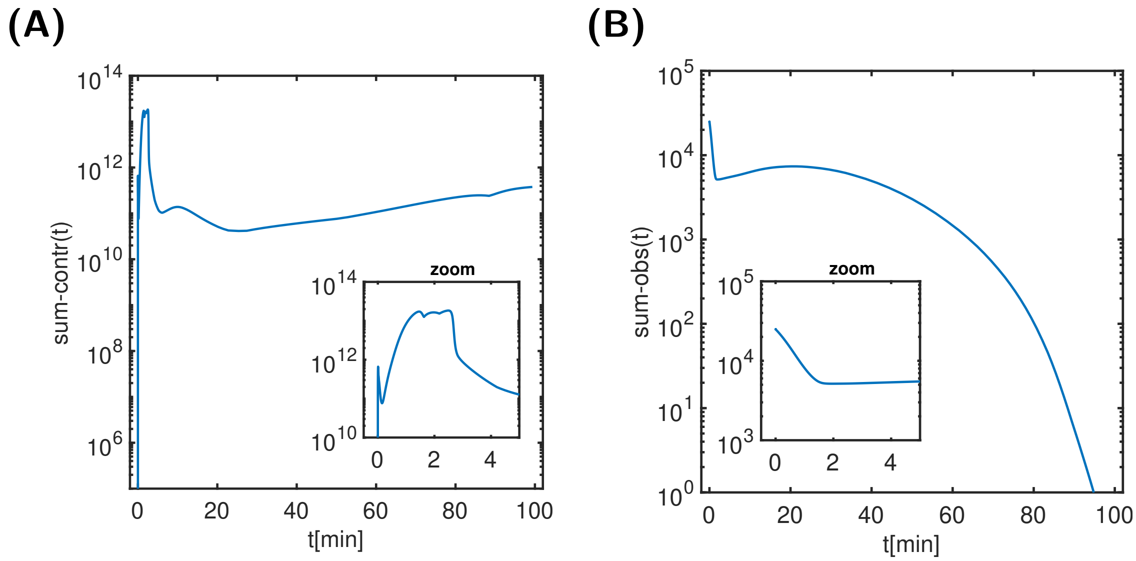

Figure III. Sum of contr- and obs-indices evolving over time. Left: Sum of contr-indices over time, showing three phases: initial sharp peak (0-0.3 min), second prolonged peak (0.3-3 min), and a slow, still incomplete recovery period (3-100 min). Right: Sum of obs-indices over time, showing three phases: initial sharp decline (0-1.5 min), marginal increase (1.5-20 min), and a strong decline (20-100 min). Note the very different scales on the y-axis.

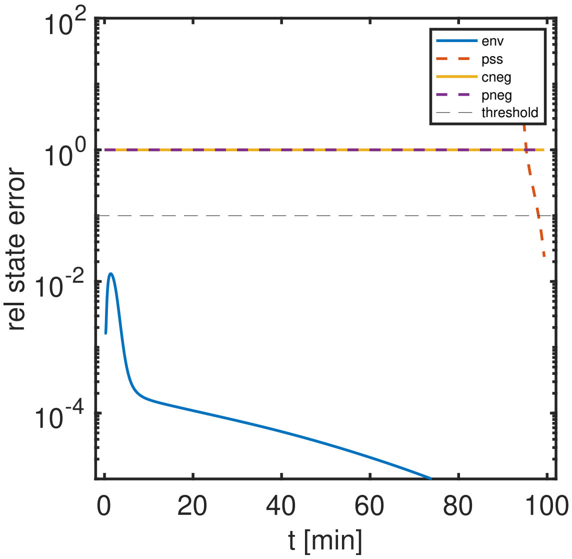

Figure IV. Relative state approximation errors for Phosphatase1

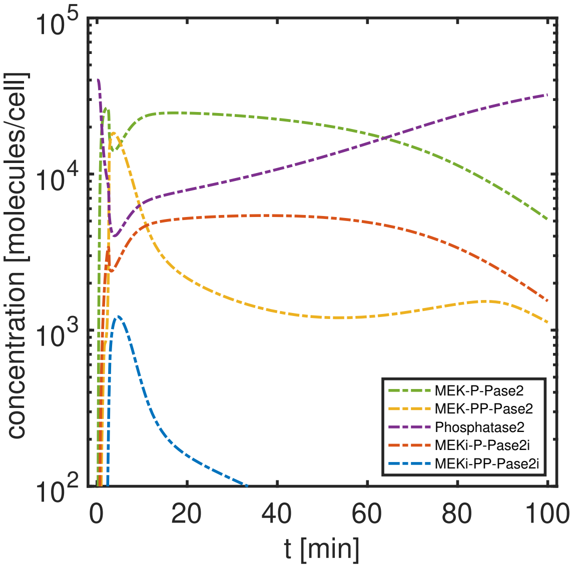

Figure V. Time course of important species related to Phosphatase2

Table I. Normalised input-response indices for the EGFR system; sorted according to their maximum

No.	Variable name	max(nir-index)
1	EGF	1
2	EGFR	4.95e-01
3	EGF-EGFR	4.76e-01
4	$(\text{EGF-EGFR*})_2$	4.57e-01
5	MEK-P	4.37e-01
6	ERK-P-MEK-PP	3.78e-01
7	ERK-PP	3.44e-01
8	Phosphatase3	3.23e-01
9	Ras-GDP	3.10e-01
10	ERK-P	2.89e-01
11	ERK	2.83e-01
12	ERK-MEK-PP	2.78e-01
13	$(\text{EGF-EGFR})_2$	2.43e-01
14	ERK-P-P'ase3	1.99e-01
15	Ras-GTP	1.99e-01
16	ERK-PP-P'ase3	1.72e-01
17	GAP	1.66e-01
18	MEK-Raf*	1.56e-01
19	MEK-PP	1.48e-01
20	$(\text{EGF-EGFR*})_2$-GAP-Shc*-Grb2-Sos-Ras-GDP	1.32e-01
21	$(\text{EGF-EGFR*})_2$-GAP-Shc*-Grb2-Sos	9.90e-02
22	ERKi-P	5.44e-02
23	MEK-P-Raf*	4.64e-02
24	MEK	3.64e-02
25	ERKi-P-P'ase3i	3.10e-02
26	Ras-GTP*	3.00e-02
27	ERKi-PP	2.88e-02
28	Shc*	2.80e-02
29	Shc	2.66e-02
30	$(\text{EGF-EGFR*})_2$-GAP-Shc*-Grb2-Sos-ERK-PP	2.38e-02
31	ERKi-PP-P'ase3i	2.09e-02
32	Raf	2.05e-02
33	$(\text{EGF-EGFR*})_2$-GAP-Shc*-Grb2-Sos-Prot	1.97e-02
34	MEKi-PP	1.76e-02
35	$(\text{EGF-EGFR*})_2$-GAP-Grb2-Sos-Ras-GDP	1.72e-02
36	$(\text{EGF-EGFRi*})_2$-GAP-Shc*-Grb2-Sos	1.66e-02
37	Phosphatase2	1.53e-02
38	$(\text{EGF-EGFR*})_2$-GAP-Shc*	1.53e-02
39	Grb2-Sos	1.47e-02
40	$(\text{EGF-EGFR*})_2$-GAP-Shc*-Grb2-Sos-Ras-GTP	1.38e-02
41	$(\text{EGF-EGFR*})_2$-GAP-Grb2-Sos	1.27e-02
42	Rasi-GTP*	1.26e-02
43	Raf-Ras-GTP	1.25e-02
44	ERKi-P-MEKi-PP	1.05e-02
45	ERKi-MEKi-PP	1.05e-02
46	$(\text{EGF-EGFR*})_2$-GAP-Shc*-Grb2-Sos-Ras-GDP-Prot	9.92e-03
47	$(\text{EGF-EGFR*})_2$-GAP	9.83e-03

Table is continued on next page

Table I – cont.

No.	Variable name	max(nir-index)
48	Prot	9.66e-03
49	$(EGF\text{-}EGFR^*)_2$-GAP-Shc*-Grb2-Sos-Ras-GTP-Prot	9.30e-03
50	MEK-PP-Pase2	9.05e-03
51	$(EGF\text{-}EGFRi^*)_2$-GAP-Shc*	8.01e-03
52	Phosphatase1	7.86e-03
53	Raf*-Pase	6.81e-03
54	$(EGF\text{-}EGFRi^*)_2$	6.57e-03
55	MEKi-Rafi*	6.34e-03
56	$(EGF\text{-}EGFR^*)_2$-GAP-Shc	6.00e-03
57	MEKi-P	5.76e-03
58	$(EGF\text{-}EGFRi^*)_2$-GAP-Shc*-Grb2-Sos-Ras-GDP	5.10e-03
59	Shc*-Grb2-Sos	4.85e-03
60	$(EGF\text{-}EGFR^*)_2$-GAP-Grb2-Sos-ERK-PP	4.04e-03
61	$(EGF\text{-}EGFRi^*)_2$-GAP	3.78e-03
62	MEKi-PP-Pase2i	2.71e-03
63	$(EGF\text{-}EGFR^*)_2$-GAP-Grb2-Sos-Ras-GTP	2.51e-03
64	MEKi-P-Pase2i	2.37e-03
65	$(EGF\text{-}EGFRi^*)_2$-GAP-Grb2-Sos	2.35e-03
66	$(EGF\text{-}EGFR^*)_2$-GAP-Shc*-Grb2	2.28e-03
67	MEK-P-Pase2	2.17e-03
68	$(EGF\text{-}EGFRi^*)_2$-GAP-Shc*-Grb2-Sos-Ras-GTP	2.16e-03
69	$(EGF\text{-}EGFR^*)_2$-GAP-Grb2	2.02e-03
70	$(EGF\text{-}EGFR^*)_2$-GAP-Grb2-Sos-Prot	1.92e-03
71	Grb2	1.83e-03
72	Rasi-GTP	1.80e-03
73	Raf*	1.73e-03
74	$(EGF\text{-}EGFR^*)_2$-GAP-Grb2-Sos-Ras-GDP-Prot	1.38e-03
75	EGFRi	1.22e-03
76	Rafi*-Pase	1.16e-03
77	$(EGF\text{-}EGFR^*)_2$-GAP-Grb2-Sos-Ras-GTP-Prot	1.06e-03
78	EGF-EGFRi	1.01e-03
79	$(EGF\text{-}EGFRi^*)_2$-GAP-Grb2-Sos-Ras-GDP	9.57e-04
80	Sos-ERK-PP	8.99e-04
81	Shc*-Grb2	7.28e-04
82	$(EGF\text{-}EGFRi^*)_2$-GAP-Shc	7.25e-04
83	$(EGF\text{-}EGFRi^*)_2$-GAP-Shc*-Grb2	7.22e-04
84	$(EGF\text{-}EGFR^*)_2$-GAP-Grb2-Prot	5.75e-04
85	$(EGF\text{-}EGFRi^*)_2$-GAP-Grb2	5.48e-04
86	$(EGF\text{-}EGFR^*)_2$-GAP-Shc*-Grb2-Prot	5.40e-04
87	Sos	5.25e-04
88	EGFi	4.86e-04
89	MEKi-P-Rafi*	4.12e-04
90	$(EGF\text{-}EGFRi^*)_2$-GAP-Grb2-Sos-Ras-GTP	2.67e-04
91	$(EGF\text{-}EGFRi^*)_2$-GAP-Shc*-Grb2-Sos-ERKi-PP	2.67e-04
92	Sos-ERKi-PP	1.20e-04
93	Rafi-Rasi-GTP	1.01e-04
94	Rafi*	7.31e-05
95	$(EGF\text{-}EGFRi)_2$	5.94e-05

Table is continued on next page

Table I – cont.

No.	Variable name	max(nir-index)
96	$(EGF\text{-}EGFRi^*)_2$-GAP-Grb2-Sos-ERKi-PP	5.44e-05
97	Proti	1.41e-08
98	$(EGF\text{-}EGFRi^*)_2$-GAP-Shc_{deg}	0
99	$(EGF\text{-}EGFRi^*)_2$-GAP_{deg}	0
100	$(EGF\text{-}EGFRi^*)_2$-GAP-Grb2-Sos-Ras_{deg}	0
101	$(EGF\text{-}EGFRi^*)_2$-GAP-Shc*-Grb2-Sos-Ras_{deg}	0
102	AUC ERK-PP	0
103	Sosi	0
104	$(EGF\text{-}EGFRi^*)_2$-GAP-Shc*-Grb2-Sos_{deg}	0
105	$(EGF\text{-}EGFRi^*)_2$-GAP-$Grb2_{deg}$	0
106	$(EGF\text{-}EGFRi^*)_2$-GAP-Grb2-Sos_{deg}	0
107	$(EGF\text{-}EGFR^*)_2$-GAP-Shc*-Grb2-Sos_{deg}	0
108	$(EGF\text{-}EGFRi^*)_2$-GAP-Shc*-$Grb2_{deg}$	0
109	$(EGF\text{-}EGFR^*)_2$-GAP-Grb2-Sos_{deg}	0
110	$(EGF\text{-}EGFRi^*)_{2deg}$	0
111	$EGFRi_{deg}$	0
112	$EGFi_{deg}$	0
