# Supplementary material for: Index analysis: An approach to understand signal transduction with application to the EGFR signalling pathway
Source: PLoS Comput Biol. 2024 Feb 5;20(2):e1011777. doi: 10.1371/journal.pcbi.1011777 (PMC10868873; doi:10.1371/journal.pcbi.1011777)
Supplement: Attachment — Submitted filename: Knoechel_etal_2023_comments2reviewers.pdf [file pcbi.1011777.s007.pdf]

Response to the reviewers' & editor's comments

Preface

We highly appreciated the constructive comments and remarks by the reviewers, as well as the opportunity to revise our manuscript. In the point-to-point response below, we have addressed all comments and remarks and revised the manuscript accordingly. We also provide a version with tracked changes. Finally, we would like to thank the reviewers for their time and consideration.

Reviewer #1 (Remarks to the Author):

In this work, Knoechel et al. introduced a new method, called 'index analysis', to study how the output of a kinetic model is affected by different types of perturbations on the state variables (model species). The authors also demonstrated the applications of this method with a simple toy model and a very complicated EGFR signaling model. The 'index analysis' method would be interesting for the field of computational systems biology. It is also potentially useful for the study of dynamical biological systems.

> We appreciate the positive feedback on the manuscript.

However, the presentation of this work needs further improvement. It is not very compelling how much insights can one learn from this method in practice. A comparison to existing sensitivity analysis methods is missing, which makes one difficult to judge the value of this method.

> We have revised the part of the introduction that reviews existing methods to puts more clearly our proposed index analysis methods into context. In brief: there are various methods to analyse, reduce and interpret complex systems biology methods, but they approach the questions "What is important?" differently. Despite this repertoire of approaches to study complex systems biology model, our means remain limited to study the *time-resolved* relevance of reactions and molecular species during signal. As concluded in [4], there is a need for "the development of suitable descriptors of time-varying transfer properties in cell signalling cascades". The presented index analysis aims to address this gap by answering the questions: At what time(s) is a molecular species relevant for the transduction of the signal? And why?
>
> Furthermore, we would like to refer to the discussion, where we did compare our approach with a control theoretical approach based on a specific type of sensitivity coefficients presented in Hornberg et al (2005), illustrated for the same EGFR model as in our analysis. The authors quantified the relevance of *reactions* based on the concept of impact control coefficients, and the importance of proteins based on a fractional change of their *total* concentration. The time-dependent output was described by three characteristics: the amplitude, duration and integral of the ERK-PP profile. Index analysis allows a complementary view on the system. It focusses on individual state variables and on time, in contrast to reactions and lumped total concentrations. In Hornberg et al (2005b), general principles that govern signal transduction are identified, with the central conclusion that collectively, kinases control amplitudes more than duration, whereas Phosphatases tend to control both. Our time-resolved index analysis of the Phosphatases of the EGRF signalling network, in particular Phosphatase3, supports this conclusion and adds an additional detail: Phosphatases might also control the time to signal onset. In addition, we identified mechanistic principles underlying the conclusion including, e.g., the stoichiometric ratio of the Phosphatases and their binding partners (see paragraph on Phosphatases1–3 in the Results). While free Phosphatase1 levels are hardly impacted by complex formation with Raf*, free Phosphatase3 levels

are reduced by three orders of magnitude; nearly 100 % of Phosphatase3 is sequestered in complex with ERK-PP for a long time.

We made the following change to the Introduction:

Lines 41-47 in the clean manuscript:
'To account for the inherently transient behaviour of signalling transduction, time-dependent sensitivity coefficients have been considered, including more robust sensitivity coefficients based on local averaging [4]. Often, however, these are subsequently converted to time-independent quantities by integration in time, facilitating comparison and accounting for delays in signal propagation that often render pointwise evaluations meaningless [3]. An alternative approach is pursued in [2] by probabilistically identifying dominant reaction paths combining topical geometry and ultradiscretization theory.'

Lines 62-82 in the clean manuscript:
'Model interpretation approaches additionally add to our understanding of complex system biology models. Topological sensitivity analysis extends classical sensitivity analysis from the impact of parameter variations to the impact of changes in model architecture [1], which has implications on the biophysical interpretation of model parameters. In [4], the dependence of parameter sensitivity on the chosen response and condition (input) has been studied shedding light on the interpretation of robustness of parameters. To dissect the relative contribution of different reaction channels with overlapping molecular species to a common readout (response), causal tracing of signal flows based on in silico molecular labelling is used in [3] to interpret the observed paradoxical activity in drug-inhibited BRAF mutant melanoma in terms of rewiring of the signalling cascade.

Despite this repertoire of approaches to study complex systems biology model, our means remain limited to study the time-resolved relevance of reactions and molecular species during signal. As concluded in [4], there is a need for "the development of suitable descriptors of time-varying transfer properties in cell signalling cascades". An attempt to study the time-resolved relevance of parameters ("At what time is a given parameter relevant?") is presented in [5], but many questions remain. Limited approaches exist to study the time-resolved relevance of molecular species. Typically, these rely on some a-priori assumed decomposition of the signalling network into modules and a focus on some key molecular species to reduce the complexity [3]. The presented index analysis aims to address this gap by answering the questions: At what time(s) is a molecular species relevant for the transduction of the signal? And why?'

Major comments:

1) It is not clear for me what are the advantages and disadvantages of the 'index analysis' compared to the existing sensitivity analysis methods. Time-dependent sensitivity analysis can be performed with control coefficients and other sensitivity analysis methods as well. As the authors have not done any comparison with previous methods. I'm not sure whether the 'index analysis' proposed in this study could reveal some insights that cannot be seen by published time-dependent sensitivity analysis methods?

Please see our answer to the reviewer's comment above. In addition, we would like to highlight the following paragraph from the Discussion:

Lines 490-509 in the clean manuscript:

The sensitivity-based input-response index is defined in terms of two sensitivity coefficients. In sensitivity analysis, local sensitivity coefficients measure the impact of changes in parameters p or initial conditions (also seen as input parameters) on a response variable x_r as a function of time [10]. They are used, e.g., for parameter sensitivity analysis or to study the robustness of signal transduction pathways. A key challenge in sensitivity analysis is to account for scale-difference and to make them invariant to transformation of units and thus comparable. A common solution is to consider scaled sensitivity coefficients

$$G_{r,i}(t) = \frac{\partial x_r(t)}{\partial p_i} \cdot \frac{p_i}{x_r(t)}, \tag{1}$$

resulting in a unitless quantity know as the logarithmic gain [7], or as control or response coefficient in metabolic control analysis [8, 9]. In signalling cascades, signals are often propagated through a series of activation steps. Activated forms temporarily rise from zero/very low concentrations to their maximum, while inactive forms almost vanish. In such a situation, the scaling above is highly problematic, since $x_r(t) \approx 0$ at initial times for active forms and $x_r(t) \approx 0$ at later times for inactive forms, resulting in ill-defined coefficients or numerical problems. Of note, metabolic control analysis has mainly been and very successfully been applied to metabolic systems in steady state, where such problems are absent. The input-response index differs in two regards: (i) it considers the integral of the first factor in eq. (1) *rather than the evaluation at a single point in time; and (ii) it scales with the controllability index of the kth state variable, rather than the factor $p/x_r(t)$ in eq.* (1). *This makes a key difference and firmly links the input-response index to a specific input, ensures invariance under internal unit transformations; and at the same time resolves the above scaling issue.*

2) The introduction to the different indices is not very straight-forward. It is not easy for the readers to follow different definition of state classification indices based on their names. Maybe the authors can invent some intuitive and easy-understandable terms. For example, equation 15 already define the index. The so-called 4 state classification indices correspond to different perturbations on a selected state variable: setting the variable with a constant value (envk); describing the dynamics of the variable with an algebraic equation (pssk); removing the reactions that contains the variable as a reactant (pnegk); setting the state variable with a value 0 (cneg, correspond to knockout). I don't understand what are the differences between pnegk and cneg? In most cases, I would expect the same results for pnegk and cneg. Is pnegk really needed? Here I would suggest the authors to simplify different types of state indices, for example, focus on only two perturbations: setting the select variable with a value of zero (cneg, KO) and non-zero value (envk). It seems more indices also causes confusions.

We thank the reviewer for this important feedback. We revised the paragraph that introduces the the state-classification indices to make it more accessible. It now highlights the common definition of all four state classification indices more clearly.

Of note, in our first analysis we did consider only (and exactly) the two indices suggested by the reviewer [11]. Working with the EGFR signalling cascade model, however, we realised that focusing on only two perturbations was not sufficient to elucidate the dynamics. In particular the need for a partial steady state index became apparent. Thus, it depends on the underlying model system, which indices will be of relevance.

Regarding the difference between pneg and cneg: Both indices are needed for a classification and thereon based reduction of state. We have adapted the illustration based on the example A $\rightleftharpoons$ B $\longrightarrow$ C to more clearly illustrate the need and difference.

We made the following change to the Methods section:

Lines 158-164 in the clean manuscript:
We introduced four state classification indices (env_k, pss_k, $pneg_k$ and $cneg_k$). Each state classification index measures the impact of a modification (mod) of the original system at time t^* on the output during the remaining time interval $[t^*, T]$. The specific type of modification defines the corresponding state classification index. For the kth state variable, the common definition of all four state classification indices and the jth output is

$$\Big[ind_k(t^*)\Big]_j = \left(\frac{1}{Z_j(t^*)}\int_{t^*}^{T}\Big(\big[(s)-(s)\big]_j\Big)^2 s\right)^{1/2} \qquad (2)$$

with $t^* \in [t_0,]$ and normalisation constant $Z_j(t^*) = \int_{t^*}^{T} \big|[(s)]_j\big|^2 s$. Here and below, (s) and (s) denote the solutions of the modified system of ODEs at time $s \geq t^*$.

Lines 170-179 in the clean manuscript:
The partial steady state index (pss_k) quantifies to what extent the kth state variable can be classified as being instantaneous in steady state; since this only affects the kth state variables, it is a partial steady state of the system. The modification of the system of ODEs at time t^* is to set the left hand side of the kth ODE to zero, resulting in a system of differential-algebraic equations (DAEs). Thus, the dynamics of the k-state variable is characterised by an algebraic equation rather than on ODE. Many numerical solvers can integrate this type of equations. Of note, we used the term 'partial steady state' to differentiate it from a quasi-steady state, which typically involves the exploitation of additional conservation laws. For further illustration and clarification, see the analysis of the enzyme kinetics model given in the Supplementary Material S5.

3) Although there are elaborated descriptions about the analysis of EGFR signaling, it is not clear for me that the state classification indices analysis results contributed to the understanding of the key molecule species as the authors stated. For example, In the paragraph line 336-351, the authors discussed the role of phosphatases 3, but what is the relevance of 'index analysis' results in this context? Isn't it already clear based on the dynamics with the reference state?

Unfortunately, it is not possible to infer from a reference dynamics the relevance of any state. The reason for this is that the reference dynamics of a state does not provide any information on how its time course impact the output. This depends on the sensitivity of the output to changes of the state (a time-dependent quantity). This sensitivity a one of the two factors that define the input-response index. The other factor quantifies to what extent the input has an impact on the time course of a state at a given time. To give an example: Even if the dynamics of a state variable is almost constant except for minor changes, one cannot say, whether these small changes do or do not have an impact on the output. The answer depends on the sensitivity of the output to small changes of this state variable (at a given point in time).

4) The authors set an important threshold value 0.1 for state classification based on indices. Without showing many examples, it is not clear how this value can be generally applied in other cases? How this value is determined?

The answer to the question motivated us to replace the single threshold δ by two thresholds, δ_y on the output (applicable to all indices), and δ_x on the states (applicable to all relative state approximation errors). In the submitted manuscript, it is $\delta = \delta_y = \delta_x$. However, distinguishing between the two thresholds allows us to more clearly discuss them. The decision tree in Figure 1 has been updated correspondingly.

The threshold δ_x on the relative state approximation error is at the modeller's choice, and depends on the purpose and requirements of the analysis. The threshold δ_y on the indices is based on our experience. For example, for the EGFR systems, the threshold value also aligned with a gap in the decay of the maxima of the normalised input-response indices, as stated in the originally submitted manuscript in lines 321-323. Matching gaps is a common strategies in choosing thresholds. To our opinion, a good choice would also result in a large fraction of the states being classified. We updated the manuscript and now provide details on the classification for the EGFR system. From a total of 112 state variables, 80 are classified, i.e, almost 3/4 of states. We consider this a high fraction. Proceeding along the decision tree, we obtain the following classification: dyn = 20, env = 2, pss = 34, cneg = 20, pneg = 2. Finally, we would like to comment that the two threshold δ_y and δ_x, however, need not be identical, and could be adapted to any specific needs.

We made the following change to the Methods section: In the corresponding paragraphs, we replaced δ by δ_x or δ_y, as mentioned above. In addition, we made the following change to the Results section:

Lines 363-371 in the clean manuscript:
In addition, we computed the state classification indices and relative state approximation errors of all states not classified as dynamic, resulting in 2 environmental, 34 in partial steady state, 41 completely negligible, and 24 partially negligible states. Of note, this classification is not unique, with the largest overlap between completely negligible and in partial steady state/partially negligible. Proceeding as in Fig. **??** *(order: dyn, env, pss, cneg, pneg) resulted in a non-overlapping classification of 2 environmental, 34 in partial steady state, 20 completely negligible, and 2 partially negligible states. In summary, the index analysis provide a classification of 80 of 112, or approximately 3/4 of the states.*

Minor comments:

1) It's not clear for me how to perform 'partial steady state index (pssk)' analysis in practice. The authors suggest to removing the ODE for the kth variable (kth ODE =0) and modify the ODE systems with DAE system. How this is implemented in practice. It will be better to illustrate it with the toy example by explicitly writing the corresponding equations of the ODE/DAE systems for different state classification indicies.

We thank the reviewer for pointing out this aspect. We have revised the Supplementary Material to make it clearer how to implement a differential algebraic system (DAE). We now include the system of ODEs and the way it needs to be modified for any of the four indices in the Supplementary Material S1; in addition, we also provide a pseudocode in S2.

For the changes made to the Supplement, please see the PDF of the Supplementary Material, Sections S1+S2.

2) The state classification indicies results are highly dependent on different parameter values and the signal input. In addition, the ODE model parameters are not always identifiable. Therefore, one should be cautious to link the sensitivity analysis to the model reduction application. This could be explained in the discussion part.

The reviewer is correct in their statement that the state classification indices (as well as the input response index) depend on the model parameters and in particular on the signal input. This is by design and an important feature. While we comment several times on the dependence on the input and its magnitude, we have now added a comment in the Methods section and in the Discussion, incl. an extension to account for parameter variations. Identifiability is related to the estimation of model *parameters*, while the indices are related to model *states*. So there is no problem with identifiability for the indices.

We made the following change to the Methods section:

In the sequel we assume that all parameter values are fixed, but the approach can be extended to distributions of parameter values accounting for variability (see Discussion).

In addition, we made the following change to the Discussion section:

Lines 605-613 in the clean manuscript:
In addition, we have extended the concept of input-response indices from a fixed reference model to an ensemble of models (based on parameter variability) representing, e.g., inter-individual or inter-cellular variability [12]. This paves the way for applications in the drug discovery and development process. In [12], we demonstrate how to use the sensitivity-based input-response index in combination with a model reduction approach detailed in [11] to reduce a large-scale system biology model of the blood coagulation cascade. The result is a small-scale pharmacokinetik/pharmacodynamic model (a Warfarin-INR model) suitable for the analysis of clinical data. Application to clinical data in a therapeutic drug monitoring context is work in progress.

3) There are some typos in the manuscript. For example,

Page 12, line 183 'partial-staedy' $\Longrightarrow$ 'partial-steady' Page 41, Fig. 2B: 'kof' $\Longrightarrow$ 'koff' Fig. 12B, same color are used for ERK-PP and Raf*

We have corrected the typos and adapted the figures—spotting these typos is kindly acknowledged.

Reviewer #2 (Remarks to the Author):

Within this manuscript, the authors extend upon their previous work adapting concepts from control theory to the characterization of biochemical networks. Additionally, they apply it to signaling networks, using a well-known EGFR pathway model as a test case.

The work appears to be very rigorous technically. (This reviewer has not taken the time to validate their derivations or their computational work. If requested by the editors, this reviewer would be happy to jump in for a deeper technical review.). The authors are, at minimum, familiar with the relevant literature, grasp the key concepts very well, and communicate everything at a high level.

We appreciate the positive feedback by the reviewer.

This reviewer has a few concerns.

1) Much of the manuscript either deals with illustrating the approach with a very simple model for illustrative purposes (Figures 2, 3, 4, 5, 6) or with illustrating the approach on a well-known, more complex model (Figures 7, 8, 9, 10, 11, 12, 13). Although walking through an approach can be helpful and informative, it can read more like a tutorial or a demonstration than a research paper. i.e. it is difficult to be excited about results from arbitrary models or flawed models; the value comes from learning about the method that could then be applied to one's system of interest. The illustrative examples are fine, but they are not necessarily convincing that this is an improvement over other methods that are available (and that the authors discuss in the introduction).

We have revised the part of the introduction that reviews existing methods to puts more clearly our proposed index analysis methods into context. In brief: there are various methods to analyse, reduce and interpret complex systems biology methods, but they approach the questions "What is important?" differently. Despite this repertoire of approaches to study complex systems biology model, our means remain limited to study the *time-resolved* relevance of reactions and molecular species during signal. As concluded in [4], there is a need for "the development of suitable descriptors of time-varying transfer properties in cell signalling cascades". The presented index analysis aims to address this gap by answering the questions: At what time(s) is a molecular species relevant for the transduction of the signal? And why?

The present manuscript addresses three points, namely (i) the introduction of the index analysis methodology and (ii) to demonstrate for small model systems, where one still has an intuition, some expectation and the ability to show something theoretically, that index analysis yields the expected results, and (iii) show that the approach is also beneficially applicable to large systems. We have chosen the EGFR system, because it is a very well known system in the field and to the readership of PLoS Computational Biology. The established knowledge on this large system allows us to critically evaluate the usefulness of the index analysis approach and to compare the results with expectation gained by previous biological insights. At the same time, there are still open questions related to the EGFR signalling system, and we demonstrate how index analysis can contribute to their answer. We feel that this is a suitable approach.

We made the following change to the Introduction:

Lines 41-47 in the clean manuscript:
'To account for the inherently transient behaviour of signalling transduction, time-dependent sensitivity coefficients have been considered, including more robust sensitivity coefficients based on local averaging [4]. Often, however, these are subsequently converted to time-independent quantities by integration in time, facilitating comparison

and accounting for delays in signal propagation that often render pointwise evaluations meaningless [3]. An alternative approach is pursued in [2] by probabilistically identifying dominant reaction paths combining topical geometry and ultradiscretization theory.'

Lines 62-82 in the clean manuscript:
'Model interpretation approaches additionally add to our understanding of complex system biology models. Topological sensitivity analysis extends classical sensitivity analysis from the impact of parameter variations to the impact of changes in model architecture [1], which has implications on the biophysical interpretation of model parameters. In [4], the dependence of parameter sensitivity on the chosen response and condition (input) has been studied shedding light on the interpretation of robustness of parameters. To dissect the relative contribution of different reaction channels with overlapping molecular species to a common readout (response), causal tracing of signal flows based on in silico molecular labelling is used in [3] to interpret the observed paradoxical activity in drug-inhibited BRAF mutant melanoma in terms of rewiring of the signalling cascade.

Despite this repertoire of approaches to study complex systems biology model, our means remain limited to study the time-resolved relevance of reactions and molecular species during signal. As concluded in [4], there is a need for "the development of suitable descriptors of time-varying transfer properties in cell signalling cascades". An attempt to study the time-resolved relevance of parameters ("At what time is a given parameter relevant?") is presented in [5], but many questions remain. Limited approaches exist to study the time-resolved relevance of molecular species. Typically, these rely on some a-priori assumed decomposition of the signalling network into modules and a focus on some key molecular species to reduce the complexity [3]. The presented index analysis aims to address this gap by answering the questions: At what time(s) is a molecular species relevant for the transduction of the signal? And why?'

2) One challenge I have with the paper is not the authors' fault, but is that the well-known EGFR model that is used as a test case has many artifacts that are not realistic. Somewhat concerning, these artificial constructs play a big role in the illustrative walk through. For example, using a computational tool to determine which of two processes is more important in a complicated dynamical system is a reasonable question to pursue. However, the discussion of SHC-dependent and SHC-independent pathways and its inference from the model is somewhat awkward to read because the model itself is not well-suited to study the biological problem. (Within the model, the EGFR homo-dimer binds to either one SHC molecule or one GRB2 molecule. In the real world, each EGFR protomer in the dimer has multiple phospho-tyrosine residues that could bind SHC1 and multiple residues that could bind GRB2, and on an EGFR homodimer, there is no limit to only one at a time across both individual EGFR proteins.) Thus, the tool may be useful for studying a model in terms of which reactions contribute to a given model output, but the example focuses on a biological question that cannot be answered with this model because the model is poorly designed to study that problem.

We thank the reviewer for pointing to this specific aspects of the EGFR model. We feel, however, that a thorough and detailed discussion of the pros and cons of the used EGFR model is beyond the scope of the present manuscript. The used EGFR model is well established and accepted in the systems biology community. It has also been foundational for the identification of promising targets in oncology see, e.g., Schoeberl et al, *Therapeutically Targeting ErbB3: A Key Node in Ligand-Induced Activation of the ErbB Receptor PI3K Axis*, Science Signalling (2009), and Schoeberl et at, *Systems biology driving drug development: from design to the clinical testing of the anti-ErbB3*

antibody seribantumab (MM-121), npj Systems Biology and Applications (2017). Finally, an entire drug discovery & development program was based on the used EGFR model (with extension to include further aspects of the ErbB receptor PI3K Axis), and a company (Merrimack) was founded.

At the same time, we feel that the systems biology community would benefit from the aspects raised by the reviewer. Hence, we would appreciate if the reviewer could provide references for their claims so that we can add a paragraph with a critical discussion of the used EGFR model.

3) Similarly, the RAS-GTP* and RAF* states of this model also seem to be artificial constructs. They do not have a clear biological equivalent. It is not clear why they were introduced (my guess is the original model included them to obtain the desired output). So discussions about RAS-GTP* and RAF* again discuss artificial aspects of the model.

Please see our answer to the previous comment.

4) My biggest concern is it does not seem that the manuscript makes a strong case that these indices are needed or offer any benefit to other techniques. The authors conclude their abstract by stating that they envision some beneficial applications (comparing healthy and diseased, model reduction, changing how models are used in drug discovery/development), but without more demonstration of the power of this method, that speculation reads very aggressive.

Aggressive comments were never our intention, neither in the abstract nor elsewhere. We removed the statement from the abstract and amended a corresponding paragraph in the discussion. In brief, we have already demonstrated the potential to reduce a large-scale quantitative systems pharmacology model to obtain a small-scale pharmacokinetic/pharmacodynamic (PK/PD) model, modelling the anti-coagulation effect of the drug Warfarin [12]. Its application to the analysis of real-world clinical data in a therapeutic drug monitoring context is closed to being submitted [13]. See also our answer to the reviewer's next but one comment.

We made the following change to the Discussion:

Lines 605-613 in the clean manuscript:
In addition, we have extended the concept of input-response indices from a fixed reference model to an ensemble of models (based on parameter variability) representing, e.g., inter-individual or inter-cellular variability [12]. This paves the way for applications in the drug discovery and development process. In [12], we demonstrate how to use the sensitivity-based input-response index in combination with a model reduction approach detailed in [11] to reduce a large-scale system biology model of the blood coagulation cascade. The result is a small-scale pharmacokinetik/pharmacodynamic model (a Warfarin-INR model) suitable for the analysis of clinical data. Application to clinical data in a therapeutic drug monitoring context is work in progress.

Suggestions for strengthening the manuscript:

One straightforward way to improve the manuscript would include some type of comparison of how these different reactions are ranked as important by these indices compared to how they would be ranked by the other methods the authors discuss in lines 36-66. Direct comparisons may not be possible – but a table or schematic that compares how the different approaches discussed would

characterize the EGFR and/or simple model, and the relative benefits and/or costs of each could be useful.

There might be a misunderstanding underlying the reviewer's comment: Index analysis does not rank reactions, but rather characterises the dynamic importance (or unimportance) of state variables as a function of time. This is very different from, e.g., sensitivity analysis, which focusses on parameter or initial value sensitivities. This was made clearer with the revision of the introduction, as mentioned above.

Most powerful would be if there is a case where the index analysis provides a biological insight that can be validated (even with existing data). That may be hard with the EGFR model (due to its artefacts) – but with the extensive EGFR kinetic signaling experimental literature, are there any things from this analysis that can be compared to existing data? Does the model help clarify anything experimentally? Or is it only useful at figuring out what is going on within the model (which here is unfortunately full of artefacts). Also helpful – if the analysis suggests new experiments, what are they? If the model is useful, stating what experiments would test model-based insights would be helpful for evaluating if the index analysis is creating new insights. Stating suggested experiments is the type of next-step that is commonly expected in manuscripts like this. (My apologies if I missed it, I only found the string "experiment" in the text once.)

The manuscript aims to provide a methodological introduction to index analysis with special focus on the sensitivity-based input-response indices and state classification indices. The application to small model systems as well as to the well known EGFR system provides credibility to the usefulness of the index analysis approach. We have added a paragraph in the Discussion on the usefulness of index analysis in application to understanding and optimising drug therapy in daily therapeutic use. The aim is to individualise Warfarin therapy for optimal anti-coagulation effect based on real-world patient data. To be more specific: There are a few small-scale empirical PK/PD models of Warfarin and its effect on the prothrombin time (a measure of the ability of the blood to clod). Although these describe the same drug therapy, the empirical models are very different, since they have been developed based on specific clinical data and statistical criteria. It is not only unclear, how they relate to each other, but also, how they related to existing, detailed and mechanistic large scale systems biology models of the blood coagulation network. This is a very unsatisfactory and critical situation, in particular in model-informed precision dosing, where models are used to optimise dose for individual patients. Using the input-response indices combined with model-reduction approaches, we derived a reduced Warfarin PK/PD model that is consistent with the large scale model [12], and able to describe real-world clinical data very well [13]. This was made clearer with the revision of the discussion, as mentioned above.

In the present manuscript, however, we have chosen the EGRF model system, since it is much more widely established in the systems biology community. Due to its larger size (twice as large as the above mentioned blood coagulation network), it is also more challenging.

Additional suggested edits:

a) Line 216 lists 'Table ??'. I think it means to say Fig 2B.

Corrected.

b) Fig 8A and Fig 8B. Both 'GAP' and 'RAS-GTP' seem to have the same color/dash line type, so they look indistinguishable in my PDF. They should be indicated with a unique color/line type combination.

> We revised the colors correspondingly.

c) Fig 12B: it states in the legend that there should be two reference trajectories in solid lines and two modified model trajectories in dashed lines, but I only see two solid lines in Fig 12B.

> We apologise for the confusion. The dashed lines are visually indistinguishable from the solid lines. We corrected the legend. We also realised that ERK-PP and Raf* used the same color and corrected it.

d) Line 391: states Figure 12A, but I believe it means to say Figure 12C.

> The reviewer is right. We corrected the typo.

e) Line 393: states Figure 12B, but I believe it means to say Figure 12D.

> The reviewer is right. We corrected the typo.

Reviewer #3 (Remarks to the Author):

Knoechel and colleagues introduce a framework that introduces several indices for the analysis of mathematical models of biological systems. They demonstrate the utility of these indices in terms of model simplification and interpretation on a small cascade example as well as a previously published, more complex model of EGFR signaling. Both aspects are relevant and timely and I am convinced that this work is an important contribution to the field. Accordingly, I would like to express my support to publish this after some minor revision

We appreciate the positive feedback on the manuscript.

1) Would be nice to describe the implementation of how these coefficients are computed numerically in a bit more detail

We thank the reviewer for pointing out the lack of information on the numerical part. We now provide a detailed description of the implementation of the ir- and state classification indices in Supplementary Material S1, incl. a pseudocode in S2. There is also a section on how to implement a new model in S3. In addition, we added a paragraph on the numerical effort of computing the indices to the Discussion.

We made the following change to the Discussion:

Lines 568-588 in the clean manuscript:
The numerical effort to compute all indices for the illustrative model systems is in the seconds, and for the EGFR signalling cascade a few hours (on a decent laptop). For the ir-index, the most demanding part is the solution of the sensitivity equation to determine the controllability index (a single simulation on $[t_0, T]$*) and the observability index (for each* t^* *a simulation on* $[t^*, T]$*); see eqs. (**??**) and Supplementary material S1&S2. We chose the number of* t^* *values to be identical to the (adaptively choosen) timepoints of the numerical scheme when integrating the reference solution. This guaranteed good approximation quality of the integral over the observability index (see eq. (**??**)) by the trapezoidal rule—in addition to smooth visual plots. Since the Jacobian of the right hand side of reaction kinetic models is typically sparse, we exploited this advantageous feature numerically (by using a sparse representation of the Jacobian matrix). For the state classification indices, the numerical effort is in simulating the modified system of ODEs (repeatedly for each* t^* *a simulation on* $[t^*, T]$*). Of note: in particular for the partial steady state index, the numerical integrator can face problems in cases, when a partial steady state assumption will result in a blow-up or when determining consistent initial conditions for the DAE system. In the first case, we simply assigned an NaN value to the corresponding index (the state would not be classified as pss anyway), while in the second case, we retried with a different initial guess of the consistent initial condition (up to three times). This approach was successfully used in all considered examples.*

If in case of very large ODE systems, e.g., when automatically generated by rules as impressively illustrated in [3], the solution of the sensitivity equation is challenging, the empirical input-response indices [11] might provide an alternative.

For the additional changes to the Supplementary Material, please consult directly the PDF, Sections S1&S2.

2) The introduction has a rather strong focus on model simplification, but there are a couple of related approaches for model interpretation that might be worth mentioning (could also be

something for the discussion):
- https://doi.org/10.1101/2023.05.02.538686
- https://doi.org/10.1038/msb.2008.74
- elasticity and other coefficients, see e.g., https://doi.org/10.1049/iet-syb.2011.0015
- https://doi.org/10.1073/pnas.1414026112
- https://doi.org/10.15252/msb.202210988
- https://doi.org/10.1101/2021.01.26.428266

We thank the reviewer for bringing up this additional angle to index analysis and providing the above references. We gladly added the point of view of model interpretation to the introduction.

We made the following change to the Introduction:

Lines 41-47 in the clean manuscript:
'To account for the inherently transient behaviour of signalling transduction, time-dependent sensitivity coefficients have been considered, including more robust sensitivity coefficients based on local averaging [4]. Often, however, these are subsequently converted to time-independent quantities by integration in time, facilitating comparison and accounting for delays in signal propagation that often render pointwise evaluations meaningless [3]. An alternative approach is pursued in [2] by probabilistically identifying dominant reaction paths combining topical geometry and ultradiscretization theory.'

Lines 62-82 in the clean manuscript:
'Model interpretation approaches additionally add to our understanding of complex system biology models. Topological sensitivity analysis extends classical sensitivity analysis from the impact of parameter variations to the impact of changes in model architecture [1], which has implications on the biophysical interpretation of model parameters. In [4], the dependence of parameter sensitivity on the chosen response and condition (input) has been studied shedding light on the interpretation of robustness of parameters. To dissect the relative contribution of different reaction channels with overlapping molecular species to a common readout (response), causal tracing of signal flows based on in silico molecular labelling is used in [3] to interpret the observed paradoxical activity in drug-inhibited BRAF mutant melanoma in terms of rewiring of the signalling cascade.

Despite this repertoire of approaches to study complex systems biology model, our means remain limited to study the time-resolved relevance of reactions and molecular species during signal. As concluded in [4], there is a need for "the development of suitable descriptors of time-varying transfer properties in cell signalling cascades". An attempt to study the time-resolved relevance of parameters ("At what time is a given parameter relevant?") is presented in [5], but many questions remain. Limited approaches exist to study the time-resolved relevance of molecular species. Typically, these rely on some a-priori assumed decomposition of the signalling network into modules and a focus on some key molecular species to reduce the complexity [3]. The presented index analysis aims to address this gap by answering the questions: At what time(s) is a molecular species relevant for the transduction of the signal? And why?'

Minor comments:

a) l8 "indices" is rather unspecific, maybe give them a better name?

We have revised the sentence to make the wording clearer.

b) eq (4) a bit surprising that u is the same dimension as x and the solution is additive. sounds like a rather specific stimulus/perturbation, so it might be worthwhile using a different symbols

The input is a delta impulse that is realised via a modification of the initial conditions (eq. (1) of the manuscript). In eq. (4), the operator $\Psi^{t,t_0}(\cdot)$ denotes the evolution (or solution) operator of an ODE system. For non-linear ODE models, it is a non-linear function of the initial condition, implying $\Psi^{t,t_0}(x_0 + u_0) \neq \Psi^{t,t_0}(x_0) + \Psi^{t,t_0}(u_0)$. We have added a corresponding comment (on the delta input) and the note that extensions to continuous inputs are possible.

c) my impression is that y_{mod} (eq 15) and x_{mod} (eq 16) are not sufficiently well introduced

We thank the reviewer for pointing out the missing definition. We have now introduced the two variables and also illustrate the modified system in the Supplementary Material S1 based on the simple reaction cycle model.

d) l222 table ref broken

Corrected.

e) l250 In contrast, give-n- ...

Corrected.

References

[1] Babtie A C, Kirk P, Stumpf M P H Topological sensitivity analysis for systems biology. PNAS 111(52):18507-18512

[2] Oscar O. Ortega and Mustafa Ozen and Blake A. Wilson and James C. Pino and Michael W. Irvin and Geena V. Ildefonso and Shawn P. Garbett and Carlos F. Lopez Probability-based mechanisms in biological networks with parameter uncertainty. bioRxiv:doi:10.1101/2021.01.26.428266

[3] Fröhlich F, Gerosa L, Muhlich J, Sorger PK. Mechanistic model of MAPK signaling reveals how allostery and rewiring contribute to drug resistance. Mol Syst Biol. 2023 Feb 10;19(2):e10988.

[4] Chen W W, Schoeberl B, Jasper P J, Niepel M, Nielsen U B, Lauffenburger D A, Sorger P K Input-output behavior of ErbB signaling pathways as revealed by a mass action model trained against dynamic data. Molecular Systems Biology. 2009; 5:239

[5] Perumal T M, Gunawan R. Understanding dynamics using sensitivity analysis: caveat and solution. BMC Systems Biology. 2011, 5:41

[6] Hornberg JJ, Binder B, Bruggeman FJ, Schoeberl B, Heinrich R, Westerhoff HV. Control of MAPK signalling: from complexity to what really matters. Oncogene. 2005;24(36):5533–5542.

[7] Blüthgen N, Legewie S. Robustness of signal transduction pathways. Cell Mol Life Sci. 2013 Jul;70(13):2259-69.

[8] Hofmeyr J H S. Metabolic control analysis in a nutshell. Proceedings of the 2nd International Conference on Systems Biology. 2001; p. 291–300.

[9] Ingalls B P, Sauro H M. Sensitivity analysis of stoichiometric networks: an extension of metabolic control analysis to non-steady state trajectories. Theoretical Biology; 2003; 222; 23–36.

[10] Zi Z. Sensitivity analysis approaches applied to systems biology models. IET Systems Biology. 2011;5(6):336.

[11] Knöchel J, Kloft C, Huisinga W. Understanding and reducing complex systems pharmacology models based on a novel input-response index. Journal of Pharmacokinetics and Pharmacodynamics. 218; 45(1):139-157

[12] Falkenhagen U, Knöchel J, Kloft C, Huisinga W. Deriving mechanism-based pharmacodynamic models by reducing quantitative systems pharmacology models: An application to warfarin. CPT: Pharmacometrics Syst. Pharmacol. 2023; 12: 432–443.

[13] Falkenhagen U, Cavallari L, Duarte J, Schmidt S, Kloft C, Huisinga W. Can quantitative systems pharmacology guide precision dosing? An illustrative warfarin-INR example. PAGE 2023; https://www.page-meeting.org/default.asp?abstract=10711.
