# Supplementary material for: Index analysis: An approach to understand signal transduction with application to the EGFR signalling pathway
Source: PLoS Comput Biol. 2024 Feb 5;20(2):e1011777. doi: 10.1371/journal.pcbi.1011777 (PMC10868873; doi:10.1371/journal.pcbi.1011777)
Supplement: Attachment — Submitted filename: Knoechel_etal_2023_comments2reviewers.pdf [file pcbi.1011777.s008.pdf]

Response to the reviewers' & editor's comments

Preface

We are delighted about the decision of the editorial board to accept the manuscript for publication. In the final manuscript, we have added a paragraph to the Discussion to account for the views of reviewer 2 (see Details below).

Reviewer #1 (Remarks to the Author):

Reviewer 1: I'm satisfied with the authors' revision.

> We appreciate the positive feedback.

Reviewer #2 (Remarks to the Author):

The point of peer review is for the authors of a manuscript to get comments from their scientific peers. The peers say what is good what is bad, and the authors are supposed to work to make improvements to the manuscript that address these concerns. When it works, peer review makes the paper better. Reviewing papers is a lot of work, it isn't particularly fun to do, and the only reason to do it as a reviewer is to help make the field better.

The paper was initially rejected with request for significant revisions. The revised paper does not seem to have significant revisions. Several important issues raised by the reviewers are dismissed, and/or addresed superficially. In one case, after the authors were directed to several problematic areas of the model they are studying and choose to focus their efforts upon, the authors do not address these issues but rather ask for references (which are easy to find, as this is 100% standard biology that was butchered in the model). Thus, the authors do not seem to understand the biology they claim their methods are here providing "answers" to questions in the field.

The revision appears to have primarily involved edits to improve clarity, to have some work done to introduction and discussion, but not to have more work that adds to solidify the body of work as in the manuscript.

One of my major concerns is that a significant part of the analysis digs into a old and flawed EGFR signaling model. The previous review provided a few clear examples of where it is flawed, including there being multiple GRB2 and SHC1 binding sites, RASGTP* and RAF* being artifacts of the model, etc. The reviewers do not address these, but rather state that others have used this model and a (failing) company has put work into this model, seeming to imply it must be good.

Particularly concerning is that much of the demonstrations focus on studying the artifacts of the model and claiming it is useful to EGFR biology.

The authors respond with an argument to the effect of "others have done it, so why can't we?" and by pointing out that the biotech Merrimack has used this model. That others do something, of course, does not mean that it's a good course of action. It also doesn't take much time on google to find that Merrimack is struggling. (This is the biotech mentioned in response to review that claims this model must be good because it is used by a drug company). It also doesn't take much work to find evidence in the literature for the things mentioned about GRB2 and SHC1 promiscuity... for example, https://www.nature.com/articles/nature12308 https://www.nature.com/articles/nature04177 and any current review on RAS activation should show that RASGTP and RASGTP* (and RAF*) are artificial constructs. The authors' inability to find experimental biology papers that are relevant to the claims of the manuscript is highly concerning. The authors continued claim that unanswered questions in EGFR biology can be answered by studying this (flawed) model with their method is also undermined by their inability to show that they understand actual EGFR biology.

Next time, the authors should spend more time pursuing the recommendations of peer review. Otherwise, just post to bioRxiv and let the work just be out there without going through peer review.

> We understand the reviewer's point of view and we have a different view. We belief that any further exchange on this topic is beyond what can be realised within the context of the reviewing process and are open for a personal discussion.
>
> In addition, we have added the following paragraph to the discussion:
>
> *During the review process, some concerns were raised about artefacts in the EGFR signalling model [1] used in our analysis that do not reflect the most recent biological understanding. Within the model, the EGFR homo-dimer bings to either one SHC or one GRB2 molecule. It has been shown, that each EGFR protomer in the dimer has multiple phospho-tyrosine residues that could bind SHC1 and multiple residues that could bind GRB2, and on the EGFR homo-dimer, there is no limit to only one at a time across both individual EGFR proteins [3, 2]. Furthermore, the biological correspondence of the RAS-GTP* and RAF* states was questioned.*

Reviewer #3 (Remarks to the Author):

The authors have adequately addressed all my concerns.

> We appreciate the positive feedback.

Editor (Remarks to the Author):

Given the reviewer's concerns, we would appreciate if the authors could, as they have offered to do, add a short paragraph to the Discussion section of the paper that addresses some potential biological limitations of the EGFR model they have used in their case study based on the suggestions of Reviewer 2.

> We have added the following paragraph to the discussion:
>
> *During the review process, some concerns were raised about artefacts in the EGFR signalling model [1] used in our analysis that do not reflect the most recent biological understanding. Within the model, the EGFR homo-dimer bings to either one SHC or one GRB2 molecule. It has been shown, that each EGFR protomer in the dimer has multiple phospho-tyrosine residues that could bind SHC1 and multiple residues that could bind GRB2, and on the EGFR homo-dimer, there is no limit to only one at a time across both individual EGFR proteins [3, 2]. Furthermore, the biological correspondence of the RAS-GTP* and RAF* states was questioned.*

References

[1] Schoeberl B, Eichler-Jonsson C, Gilles E D, Müller G. Computational modeling of the dynamics of the MAP kinase cascade activated by surface and internalised EGF receptors. Nature Biotechnology. 2002; 20(4): 370–375.

[2] Zheng Y, Zhang C, Croucher D R, Soliman M A, St-Denis N, Pasculescu A, Taylor L, Tate S A, Rod Hardy W, Colwill K, Dai A Y, Bagshaw R, Dennis J W, Gingras A C, Daly R J, Pawson T Temporal regulation of EGF signalling networks by the scaffold protein Shc1 Nature 2013; 499(7457): 166–171

[3] Jones R B, Gordus A, Krall J A, MacBeath G A quantitative protein interaction network for the ErbB receptors using protein microarrays Nature 2006; 439(7073): 168–174
